# Analysis of circular genome rearrangement by fusions, fissions and block-interchanges

**DOI:** 10.1186/1471-2105-7-295

**Published:** 2006-06-12

**Authors:** Chin Lung Lu, Yen Lin Huang, Tsui Ching Wang, Hsien-Tai Chiu

**Affiliations:** 1 Department of Biological Science and Technology, National Chiao Tung University, Hsinchu 300, ROC, Taiwan; 2 Department of Computer Science, National Tsing Hua University, Hsinchu 300, ROC, Taiwan

## Abstract

**Background:**

Analysis of genomes evolving via block-interchange events leads to a combinatorial problem of sorting by block-interchanges, which has been studied recently to evaluate the evolutionary relationship in distance between two biological species since block-interchange can be considered as a generalization of transposition. However, for genomes consisting of multiple chromosomes, their evolutionary history should also include events of chromosome fusions and fissions, where fusion merges two chromosomes into one and fission splits a chromosome into two.

**Results:**

In this paper, we study the problem of genome rearrangement between two genomes of circular and multiple chromosomes by considering fusion, fission and block-interchange events altogether. By use of permutation groups in algebra, we propose an O
 MathType@MTEF@5@5@+=feaafiart1ev1aaatCvAUfKttLearuWrP9MDH5MBPbIqV92AaeXatLxBI9gBamrtHrhAL1wy0L2yHvtyaeHbnfgDOvwBHrxAJfwnaebbnrfifHhDYfgasaacH8akY=wiFfYdH8Gipec8Eeeu0xXdbba9frFj0=OqFfea0dXdd9vqai=hGuQ8kuc9pgc9s8qqaq=dirpe0xb9q8qiLsFr0=vr0=vr0dc8meaabaqaciaacaGaaeqabaWaaeGaeaaakeaaimaacqWFoe=taaa@383D@(*n*^2^) time algorithm to efficiently compute and obtain a minimum series of fusions, fissions and block-interchanges required to transform one circular multi-chromosomal genome into another, where *n *is the number of genes shared by the two studied genomes. In addition, we have implemented this algorithm as a web server, called FFBI, and have also applied it to analyzing by gene orders the whole genomes of three human *Vibrio *pathogens, each with multiple and circular chromosomes, to infer their evolutionary relationships. Consequently, our experimental results coincide well with our previous results obtained using the chromosome-by-chromosome comparisons by landmark orders between any two *Vibrio *chromosomal sequences as well as using the traditional comparative analysis of 16S rRNA sequences.

**Conclusion:**

FFBI is a useful tool for the bioinformatics analysis of circular and multiple genome rearrangement by fusions, fissions and block-interchanges.

## Background

For the past two decades, genome rearrangements have been studied and can be modelled to learn more about the evolution of mitochondrial, chloroplast, viral, bacterial and mammalian genomes [1]. To evaluate the evolutionary distance between two related genomes in gene order, various rearrangement events acting on genes within or among chromosomes have been proposed, such as reversals (also known as inversions) [1-10], transpositions [11,12], block-interchanges [13-15], translocations [16,17], and fusions and fissions [18,19]. Most genome rearrangement studies in computation involve the issue of solving the combinatorial problem to find an optimal series of rearrangements required to transform one genome into another.

Recently, the study on the genome rearrangement by block-interchanges has increasingly drawn great attention, since the block-interchange event is a generalization of transposition and, currently, its computational models measuring the genetic distance are more tractable than those modeled by transposition. Christie [13] first introduced the concept of block-interchange, affecting a chromosome by swapping two non-intersecting blocks containing any number of consecutive genes. Block-interchange can be considered as a generalization of transposition, since any exchanged blocks via transposition must be contiguous in a chromosome, whereas those via block-interchange need not be. As a matter of fact, the occurrence of an exchange of two non-contiguous blocks has been suggested in the previous studies related to the biological processes of bacterial replication [[20], and references therein]. Christie also proposed an O
 MathType@MTEF@5@5@+=feaafiart1ev1aaatCvAUfKttLearuWrP9MDH5MBPbIqV92AaeXatLxBI9gBamrtHrhAL1wy0L2yHvtyaeHbnfgDOvwBHrxAJfwnaebbnrfifHhDYfgasaacH8akY=wiFfYdH8Gipec8Eeeu0xXdbba9frFj0=OqFfea0dXdd9vqai=hGuQ8kuc9pgc9s8qqaq=dirpe0xb9q8qiLsFr0=vr0=vr0dc8meaabaqaciaacaGaaeqabaWaaeGaeaaakeaaimaacqWFoe=taaa@383D@(*n*^2^) time algorithm, where *n *is the number of genes, to solve the so-called *block-interchange distance problem *that is to find a minimum series of block-interchanges for transforming one linear chromosome into another. Later, we [14] designed a simpler algorithm for solving the block-interchange problem on linear or circular chromosomes with time-complexity of O
 MathType@MTEF@5@5@+=feaafiart1ev1aaatCvAUfKttLearuWrP9MDH5MBPbIqV92AaeXatLxBI9gBamrtHrhAL1wy0L2yHvtyaeHbnfgDOvwBHrxAJfwnaebbnrfifHhDYfgasaacH8akY=wiFfYdH8Gipec8Eeeu0xXdbba9frFj0=OqFfea0dXdd9vqai=hGuQ8kuc9pgc9s8qqaq=dirpe0xb9q8qiLsFr0=vr0=vr0dc8meaabaqaciaacaGaaeqabaWaaeGaeaaakeaaimaacqWFoe=taaa@383D@(*δn*), where *δ *is the the minimum number of block-interchanges required for the transformation and can be calculated in O
 MathType@MTEF@5@5@+=feaafiart1ev1aaatCvAUfKttLearuWrP9MDH5MBPbIqV92AaeXatLxBI9gBamrtHrhAL1wy0L2yHvtyaeHbnfgDOvwBHrxAJfwnaebbnrfifHhDYfgasaacH8akY=wiFfYdH8Gipec8Eeeu0xXdbba9frFj0=OqFfea0dXdd9vqai=hGuQ8kuc9pgc9s8qqaq=dirpe0xb9q8qiLsFr0=vr0=vr0dc8meaabaqaciaacaGaaeqabaWaaeGaeaaakeaaimaacqWFoe=taaa@383D@(*n*) time in advance. We also demonstrated that block-interchange events play a significant role in the genetic evolution of bacterial (*Vibrio*) species. Very recently, based on this algorithm, we have further implemented a tool, called ROBIN, for analyzing the rearrangements of gene orders via block-interchanges between two linear/circular chromosomal genomes [15]. Not only gene-order data but also sequence data are allowed to be input into the ROBIN system. If the input is the sequence data, ROBIN can automatically search for the common homologous/conserved regions shared by all input sequences.

It should be noted that the above block-interchange studies were dedicated to genomes containing only one chromosome (i.e., uni-chromosomal genomes) for evaluating their evolutionary relationships. However, for biological species with different numbers of chromosomes, the evolutionary history must also consider events of chromosomal fusions and fissions. A *fusion *occurs when two chromosomes merge into one and a *fission *takes place when a chromosome splits into two. The reason is that different chromosomes may as well exchange their genetic material with each other and, moreover, this exchange can only be achieved via inter-chromosomal operations such as fusions and fissions, instead of intra-chromosomal operations like block-interchanges. Hence, it is worthwhile to study genome rearrangements considering fusions, fissions and block-interchanges altogether. In this paper, we solve such a genome rearrangement problem by designing an efficient algorithm to compute and obtain a minimum series of all the events involving fusions, fissions and block-interchanges that are required to transform one circular multi-chromosomal genome into another, when both have the same set of genes without repeats. Although most eukaryotic genomes are linear, most prokaryotic (e.g., bacterial) genomes are circular and some of them consist of multiple circular chromosomes and large plasmids^1^. For example, some important bacterial pathogens like *Brucella*, *Burkholderia*, *Leptospira *and *Vibrio *species fall into this category. Notably, our approach is based on permutation group in algebra, instead of breakpoint graph, a commonly used approach in the study of genome rearrangement.

Recently, Yancopoulos *et al*. [21] used breakpoint graph to design an algorithm to solve a genome rearrangement problem in which the considered reversals, translocations (including fusions and fissions) and block-interchanges were given different weights. Unfortunately, their algorithm cannot be applied to solving our problem in which the events we considered are unweighted, because a series of weighted events with minimum weights in total may not be a minimum series of unweighted events, provided the events are given different weights.

## Results and discussion

Based on Algorithm Sorting-by-ffbi developed in this study, we have implemented a web server, called FFBI [22], in which biologists or scientists in genomics can conduct comprehensive analyses of circular genome rearrangements by fusions, fissions and block-interchanges for their scientific interests and needs. Furthermore, we used this web server to conduct the rearrangement analyses on the whole genomes of three pathogenic *Vibrio *species, including *V. vulnificus*, *V. parahaemolyticus *and *V. cholerae*, to infer their evolutionary relationships.

Each of these three *Vibrio *pathogens consists of two circular chromosomes, and all their genomic sequences have recently been reported in GenBank with protein-coding genes annotated (see Table 1 for their sequence information). As annotated in GenBank (as of April 2006), the genomes of *V. vulnificus*, *V. parahaemolyticus *and *V. cholerae *contain 5098, 4992 and 4008 genes, respectively. From these protein-coding genes, we identified a total of 2393 (one-to-one) orthologous genes that are physically located in different positions on the chromosomes (see the Method section for construction of orthologous genes). Inevitably, there can be a high possibility that some genes with mis-annotated or uncertain protein functions are included in the genome annotation data. We therefore used only those authentic genes whose protein functions are not annotated as hypothetical or putative proteins, or are conserved and not poorly characterized (e.g., not those genes with only general function prediction or unknown function) in the NCBI COGs [23] database of orthologous genes. As a result, there are 1274 authentic orthologous genes in total remained for the further study of genome rearrangement. The relative orders of these orthologous genes along chromosomes, as well as the annotated COGs of their coding proteins, are detailed in the web site of our server.

For each pair of these pathogenic *Vibrio *species, the variation in their gene orders has suggested that the genome rearrangement events have occurred and their genomes are closely related in evolution. To evaluate the contribution of fusions, fissions and block-interchanges to these observed rearrangements, we used the server developed in this study to compute the rearrangement distance between the gene orders of any two *Vibrio *genomes. Consequently, as shown in Table 2, the calculated rearrangement distance between *V. vulnificus *and *V. parahaemolyticus *is smaller than that between *V. vulnificus *and *V. cholerae *and that between *V. parahaemolyticus *and *V. cholerae*, suggesting that *V. vulnificus *is closer to *V. parahaemolyticus *than to *V. cholerae *in evolutionary relationship. Intriguingly, this result of genome-wide experiment well coincides with those we obtained in the previous chromosome-wide experiments [14,15]. Recall that our previous experiments were conducted in a chromosome-by-chromosome style of computing the block-interchange distance between the landmark orders of any two large/small *Vibrio *chromosomes, where the used landmarks are the maximal unique matches (MUMs) or the locally collinear blocks (LCBs) that are commonly shared by three large/small *Vibrio *chromosomes.

In fact, the evolutionary relationships of the three pathogenic *Vibrio *species revealed in our experiment of analyzing their genome rearrangements also confirms that obtained by the biological community on the basis of the traditional comparative analysis of 16S rRNA gene sequences [24-26]. For confirmation, we here repeated this comparative analysis as follows. The 16S rRNA gene sequences of three Vibrios were first aligned using the Clustal W program [27], from which the distance matrix (as shown in Table 3) was then estimated by the algorithm of Kimura's two-parameter model in PHYLIP package [28].

## Conclusion

In this paper, we studied the genome rearrangement problem between circular genomes with multiple chromosomes by simultaneously considering fusion, fission and block-interchange events. We have shown in the Method section that an optimal series of events required to transform one genome into another can be obtained in a canonical order such that all fusions come before all block-interchanges, which come before all fissions. Based on this property as well as the concept of permutation groups in algebra, we have successfully designed an O
 MathType@MTEF@5@5@+=feaafiart1ev1aaatCvAUfKttLearuWrP9MDH5MBPbIqV92AaeXatLxBI9gBamrtHrhAL1wy0L2yHvtyaeHbnfgDOvwBHrxAJfwnaebbnrfifHhDYfgasaacH8akY=wiFfYdH8Gipec8Eeeu0xXdbba9frFj0=OqFfea0dXdd9vqai=hGuQ8kuc9pgc9s8qqaq=dirpe0xb9q8qiLsFr0=vr0=vr0dc8meaabaqaciaacaGaaeqabaWaaeGaeaaakeaaimaacqWFoe=taaa@383D@(*n*^2^) time algorithm to obtain the minimum number of fusion, fission and block-interchange events for the transformation and also to generate an optimal scenario of the required rearrangement events. In addition, we have practically implemented this algorithm as a web server and applied it to analyzing by gene orders the whole genomes of three human *Vibrio *pathogens to infer their evolutionary relationships. As a consequence, our experimental results well coincide with the previous results obtained using the chromosome-by-chromosome comparisons by landmark orders between any two *Vibrio *chromosomal sequences as well as using the traditional comparative analysis of 16S rRNA sequences. The algorithm, however, should not be applied on linear multi-chromosomal genomes, because as mentioned in the Method section, it is not always possible to have an optimal scenario in a canonical order for linear genomes. Further studies in genome rearrangement can still be pursued to solve the problem for linear multi-chromosomal genomes.

## Methods

### Permutations versus genome rearrangements

**Figure 1:**
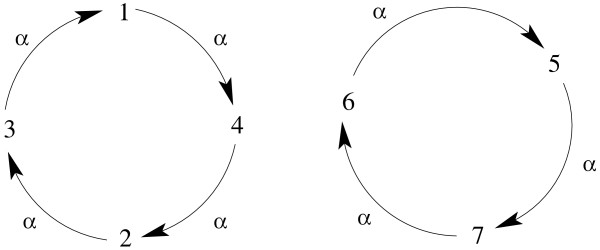
The illustration of a permutation *α *= (1, 4, 2, 3) (5, 7, 6) meaning that *α*(1) = 4, *α*(2) = 3, *α*(3) = 1, *α*(4) = 2, *α*(5) = 7, *α*(7) = 6 and *α*(6) = 5.

In group theory, a *permutation *is defined to be a one-to-one mapping from a set *E *= {1, 2, ..., *n*} into itself, where *n *is some positive integer. For example, we may define a permutation *α *of the set {1, 2, 3, 4, 5, 6, 7} by specifying *α*(1) = 4, *α*(2) = 3, *α*(3) = 1, *α*(4) = 2, *α*(5) = 7, *α*(7) = 6 and *α*(6) = 5. The above mapping can be expressed using a *cycle notation *as illustrated in Figure 1 and simply denoted by *α *= (1, 4, 2, 3) (5, 7, 6). A cycle of length *k*, say (*a*_1_, *a*_2_, ..., *a*_*k*_), is simply called *k-cycle *and can be rewritten as (*a*_*i*_, *a*_*i*+1_, ..., *a*_*k*_, *a*_1_, ..., *a*_*i*-1_), where 2 ≤ *i *<*k*, or (*a*_*k*_, *a*_1_, *a*_2_, ..., *a*_*k*-1_). Any two cycles are said to be *disjoint *if they have no element in common. In fact, any permutation, say *α*, can be written in a unique way as the product of disjoint cycles, which is called the *cycle decomposition *of *α*, if we ignore the order of the cycles in the product [29]. Usually, a cycle of length one in *α *is not explicitly written and its element, say *x*, is said to be *fixed *by *α *since *α*(*x*) = *x*. Especially, the permutation whose elements are all fixed is called an *identity permutation *and is denoted by **1 **(i.e., **1 **= (1) (2) ... (*n*)).

Given two permutations *α *and *β *of *E*, the *composition *(or *product*) of *α *and *β*, denoted by *αβ*, is defined to be a permutation of *E *with *αβ*(*x*) = *α*(*β*(*x*)) for all *x *∈ *E*. For instance, if we let *E *= {1, 2, 3, 4, 5, 6}, *α *= (2, 3) and *β *= (2, 1, 5, 3, 6, 4), then *αβ *= (2, 1, 5) (3, 6, 4). If *α *and *β *are disjoint cycles, then *αβ *= *βα*. The *inverse *of *α *is defined to be a permutation, denoted by *α*^-1^, such that *αα*^-1 ^= *α*^-1^*α *= **1**. If a permutation is expressed by the product of disjoint cycles, then its inverse can be obtained by just reversing the order of the elements in each cycle. For example, if *α *= (2, 1, 5) (3, 6, 4), then *α*^-1 ^= (5, 1, 2) (4, 6, 3). Clearly, *α*^-1 ^= *α *if *α *is a 2-cycle.

Meidanis and Dias [19,30] first noted that each cycle of a permutation may represent a circular chromosome of a genome with each element of the cycle corresponding to a gene, and the order of the cycle corresponding to the gene order of the chromosome. Figure 1, for example, shows a genome with two circular chromosomes, one represented by (1, 4, 2, 3) and the other by (5, 7, 6). Moreover, they observed that global evolutionary events, such as fusions and fissions (respectively, transpositions), correspond to the composition of a 2-cycle (respectively, 3-cycles) and the permutation representing a genome. For instance, let *α *be any permutation whose cycle decomposition is *α*_1_*α*_2_ ... *α*_*r*_. If *ρ *= (*x*, *y*) is a 2-cycle and *x *and *y *are in the different cycles of *α*, say *α*_*p *_= (*a*_1 _≡ *x*, *a*_2_, ..., *a*_*i*_) and *α*_*q *_= (*b*_1 _≡ *y*, *b*_2_, ..., *b*_*j*_) where 1 ≤ *p*, *q *≤ *r*, then in the composition *ρα*, *α*_*p *_and *α*_*q *_are joined into a cycle (*a*_1_, *a*_2_, ..., *a*_*i*_, *b*_1_, *b*_2_, ..., *b*_*j*_), i.e., *ρ *is a fusion event affecting on *α *(and also called a *join operation *of *α *here). If *ρ *= (*x*, *y*) is a 2-cycle and *x *and *y *are in the same cycle of *α*, say *α*_*p *_= (*a*_1 _≡ *x*, *a*_2_, ..., *a*_*i *_≡ *y*, *a*_*i*+1_, ..., *a*_*j*_) where 1 ≤ *p *≤ *r*, then in the composition *ρα*, this cycle *α*_*p *_is broken into two disjoint cycles (*a*_1_, *a*_2_, ..., *a*_*i*-1_) and (*a*_*i*_, *a*_*i*+1_, ..., *a*_*j*_), i.e., *ρ *is a fission event affecting on *α *(and also called a *split operation *of *α *here). If *ρ *= (*x*, *y*, *z*) is a 3-cycle and *x*, *y *and *z *are in the same cycle of *α*, say *α*_*p *_= (*a*_1 _≡ *x*, *a*_2_, ..., *a*_*i*_, *b*_1 _≡ *y*, *b*_2_, ..., *b*_*j*_, *c*_1 _≡ *z*, *c*_2_, ..., *c*_*k*_) where 1 ≤ *p *≤ *r*, then in the composition *ρα*, the cycle *α*_*p *_becomes (*a*_1_, *a*_2_, ..., *a*_*i*_, *c*_1_, *c*_2_, ..., *c*_*k*_, *b*_1_, *b*_2_, ..., *b*_*j*_), i.e., *ρ *is a transposition event affecting on *α*.

Recently, Lin *et al. *[14] further observed that a block-interchange event affecting on *α *corresponds to the composition of two 2-cycles, say *ρ*_1 _and *ρ*_2_, and *α *under the condition that *ρ*_1 _is a split operation of *α *and *ρ*_2 _is a join operation of *ρ*_1_*α*. More clearly, let *α*_*p *_= (*a*_1_, *a*_2_,..., *a*_*k*_) be a cycle of *α*, *ρ*_1 _= (*a*_1_, *a*_*i*_) and *ρ*_2 _= (*a*_*h*_, *a*_*j*_), where 1 <*i *≤ *k*,1 ≤ *h *≤ *i *- 1 and *i *≤ *j *≤ *k*. Then *ρ*_2_*ρ*_1_*α *is the resulting permutation by exchanging the blocks [*a*_*h*_, *a*_*i*-1_] and [*a*_*j*_, *a*_*k*_] of *α*_*p*_. That is, *ρ*_2_*ρ*_1 _is a block-interchange event affecting on *α *by swapping [*a*_*h*_, *a*_*i*-1_] and [*a*_*j*_, *a*_*k*_], two non-intersecting blocks in *α*.

As discussed above, any series of fusions, fissions and block-interchanges required to transform one circular multi-chromosomal genome *α *into another *I *can be expressed by a product of 2-cycles, say *ρ*_*k*_*ρ*_*k*-1_...*ρ*_1_, such that *ρ*_*k*_*ρ*_*k*-1_...*ρ*_1_*α *= *I *(hence, *ρ*_*k*_*ρ*_*k*-1_...*ρ*_1 _= *Iα*^-1^). This property implies that *Iα*^-1 ^contains all information that can be utilized to derive *ρ*_1_, *ρ*_2_,...,*ρ*_*k*_for transforming *α *into *I*.

It is well known that every permutation can be written as a product of 2-cycles. For example, (1, 2, 3, 4) = (1, 4) (1, 3) (1, 2). However, there are many ways of expressing a permutation *α *as a product of 2-cycles [29]. Given a permutation *α*, let *f*(*α*) denote the number of the disjoint cycles in the cycle decomposition of *α*. Notice that *f*(*α*) counts also the non-expressed cycles of length one. For example, if *α *= (1, 5) (2, 4) is a permutation of *E *= {1, 2,..., 5}, then *f*(*α*) = 3, instead of *f*(*α*) = 2, since *α *= (1, 5) (2, 4) (3). Then the following lemma shows the lower bound of the number of 2-cycles in any product of 2-cycles of a permutation.

**Lemma 1 ***Let α be an arbitrary permutation of E *= {1, 2,..., *n*}. *If α can be expressed as a product of m 2-cycles, say α *= *α*_1_*α*_2_...*α*_*m *_with each *α*_*i*_* being 2-cycle, then m *≥ *n *- *f*(*α*).

*Proof*. We prove this lemma by induction on *m*. The lemma is true if *m *= 0, since *α *= **1 **then, meaning that *f*(*α*) = *n*, and hence *m *= *n *- *f*(*α*) = 0. Suppose now that the lemma holds for any permutation that can expressed as a product of less than *m *2-cycles, where *m *> 0. Let *α*' = *α*_2_*α*_3_, ...,*α*_*m*_. Then by the induction hypothesis, we have *m *- 1 ≥ *n *- *f*(*α*'). Since *α *= *α*_1_*α*' and *α*_1 _is a 2-cycle, *α*_1 _operates on *α*' either as a fusion by joining two cycles of *α*' into one cycle (i.e., *f*(*α*) = *f*(*α*') - 1) or as a fission by splitting one cycle of *α*' into two cycles (i.e., *f*(*α*) = *f*(*α*') + 1). Whichever *α*_1 _operates on *α*', we have *f*(*α*) ≥ *f*(*α*') - 1. As a result, *m *= (*m *- 1) + 1 ≥ *n *- *f*(*α*') + 1 = *n *- (*f*(*α*') - 1) ≥ *n *- *f*(*α*).     □

### Optimal scenario in canonical order

As mentioned previously, each circular multi-chromosomal genome with *n *genes can be expressed by a permutation of *E *= {1, 2,..., *n*}. Given two such genomes *G*_1 _and *G*_2 _over the same gene set *E*, the *genome rearrangement distance *between *G*_1 _and *G*_2_, denoted by *d*(*G*_1_,*G*_2_), is defined to be the minimum number of events needed to transform *G*_1 _into *G*_2_, where the events allowed to take place are fusions, fissions and block-interchanges. In this section, we shall show that there is an optimal series of events required to transform *G*_1 _into *G*_2 _such that all fusions come prior to all block-interchanges, which come before all fissions. Here, such an optimal scenario of genome rearrangements is referred as in *canonical order*.

**Lemma 2 ***d*(*G*_1_, *G*_2_) = *d*(*G*_2_, *G*_1_).

*Proof*. Let Φ = <*σ*_1_, *σ*_2_,...,*σ*_*δ*_> be an optimal series of events required to transform *G*_1 _into *G*_2_. Clearly, Φ' = <*σ*_*δ*_, *σ*_*δ*-1_,...,*σ*_1_> is an optimal series of events for transforming *G*_2 _into *G*_1 _by reversing the role of every event *σ*_*i*_, where 1 ≤ *i *≤ *δ*, such that *σ*_*i*_ is a fission (respectively, fusion) in Φ' if *σ*_*i *_is a fusion (respectively, fission) in Φ.

**Lemma 3 ***There is an optimal series of events required to transform G*_1 _*into G*_2 _*such that every fission occurs after every fusion and block-interchange*.

*Proof*. Let Φ = <*σ*_1_, *σ*_2_,...,*σ*_*δ*_> be an optimal series of events needed to transform *G*_1 _into *G*_2_. Of course, if every fission occurs after every fusion and block-interchange in Φ, then the proof is done. Now, suppose that not every fission occurs after every fusion or block-interchange in Φ. Then let *i *be the largest index in Φ such that *σ*_*i *_is a fission preceding *σ*_*i*+1 _that is either a fusion or a block-interchange. We can then obtain a new optimal series Φ' = <*σ*_1_,...,*σ*_*i*-1_, σ′i
 MathType@MTEF@5@5@+=feaafiart1ev1aaatCvAUfKttLearuWrP9MDH5MBPbIqV92AaeXatLxBI9gBaebbnrfifHhDYfgasaacH8akY=wiFfYdH8Gipec8Eeeu0xXdbba9frFj0=OqFfea0dXdd9vqai=hGuQ8kuc9pgc9s8qqaq=dirpe0xb9q8qiLsFr0=vr0=vr0dc8meaabaqaciaacaGaaeqabaqabeGadaaakeaaiiGacuWFdpWCgaqbamaaBaaaleaaieGacqGFPbqAaeqaaaaa@300F@, σ′i+1
 MathType@MTEF@5@5@+=feaafiart1ev1aaatCvAUfeBSjuyZL2yd9gzLbvyNv2CaerbwvMCKfMBHbqedmvETj2BSbqee0evGueE0jxyaibaieIgFLIOYR2NHOxjYhrPYhrPYpI8F4rqqrFfpeea0xe9Lq=Jc9vqaqpepm0xbbG8FasPYRqj0=yi0lXdbba9pGe9qqFf0dXdHuk9fr=xfr=xfrpiWZqaaeaabiGaaiaacaqabeaabeqacmaaaOqaaGGaciqb=n8aZzaafaWaaSbaaSqaamXvP5wqonvsaeHbhL2B2fMBULgic92BRbaceiGae4xAaKgceaGae03kaSIae0xmaedabeaaaaa@4216@, *σ*_*i*+2_,...,*σ*_*δ*_> to transform *G*_1 _into *G*_2 _such that σ′i
 MathType@MTEF@5@5@+=feaafiart1ev1aaatCvAUfKttLearuWrP9MDH5MBPbIqV92AaeXatLxBI9gBaebbnrfifHhDYfgasaacH8akY=wiFfYdH8Gipec8Eeeu0xXdbba9frFj0=OqFfea0dXdd9vqai=hGuQ8kuc9pgc9s8qqaq=dirpe0xb9q8qiLsFr0=vr0=vr0dc8meaabaqaciaacaGaaeqabaqabeGadaaakeaaiiGacuWFdpWCgaqbamaaBaaaleaaieGacqGFPbqAaeqaaaaa@300F@ is a fusion or a block-interchange and σ′i+1
 MathType@MTEF@5@5@+=feaafiart1ev1aaatCvAUfeBSjuyZL2yd9gzLbvyNv2CaerbwvMCKfMBHbqedmvETj2BSbqee0evGueE0jxyaibaieIgFLIOYR2NHOxjYhrPYhrPYpI8F4rqqrFfpeea0xe9Lq=Jc9vqaqpepm0xbbG8FasPYRqj0=yi0lXdbba9pGe9qqFf0dXdHuk9fr=xfr=xfrpiWZqaaeaabiGaaiaacaqabeaabeqacmaaaOqaaGGaciqb=n8aZzaafaWaaSbaaSqaamXvP5wqonvsaeHbhL2B2fMBULgic92BRbaceiGae4xAaKgceaGae03kaSIae0xmaedabeaaaaa@4216@ is a fission, as discussed below. Suppose that *σ*_*i*_ splits a chromosome *α *into *α*_1 _and *α*_2_. If *σ*_*i*+1 _is a fusion, then we assume that it joins two chromosomes *β*_1 _and *β*_2_into *β*; otherwise, if *σ*_*i*+1 _is a block-interchange, then assume that it affects *β*_1 _such that *β*_1 _becomes *β *through a block-interchange. Clearly, if neither *β*_1 _nor *β*_2 _is created by *σ*_*i*_, then the desired series Φ' is obtained by swapping *σ*_*i*_ and *σ*_*i*+1 _in Φ (i.e., σ′i
 MathType@MTEF@5@5@+=feaafiart1ev1aaatCvAUfKttLearuWrP9MDH5MBPbIqV92AaeXatLxBI9gBaebbnrfifHhDYfgasaacH8akY=wiFfYdH8Gipec8Eeeu0xXdbba9frFj0=OqFfea0dXdd9vqai=hGuQ8kuc9pgc9s8qqaq=dirpe0xb9q8qiLsFr0=vr0=vr0dc8meaabaqaciaacaGaaeqabaqabeGadaaakeaaiiGacuWFdpWCgaqbamaaBaaaleaaieGacqGFPbqAaeqaaaaa@300F@ = *σ*_*i*+1 _and σ′i+1
 MathType@MTEF@5@5@+=feaafiart1ev1aaatCvAUfeBSjuyZL2yd9gzLbvyNv2CaerbwvMCKfMBHbqedmvETj2BSbqee0evGueE0jxyaibaieIgFLIOYR2NHOxjYhrPYhrPYpI8F4rqqrFfpeea0xe9Lq=Jc9vqaqpepm0xbbG8FasPYRqj0=yi0lXdbba9pGe9qqFf0dXdHuk9fr=xfr=xfrpiWZqaaeaabiGaaiaacaqabeaabeqacmaaaOqaaGGaciqb=n8aZzaafaWaaSbaaSqaamXvP5wqonvsaeHbhL2B2fMBULgic92BRbaceiGae4xAaKgceaGae03kaSIae0xmaedabeaaaaa@4216@ = *σ*_*i*_). If both *β*_1 _and *β*_2 _are created by *σ*_*i*_, then the net rearrangement of *σ*_*i*_ (a split operation) followed by *σ*_*i*+1 _(a joint operation) either has no effect on *α *or becomes a block-interchange affecting *α*. By removing *σ*_*i *_and *σ*_*i*+1 _from Φ or replacing them with an extra block-interchange, we thus obtain a new optimal series of the events transforming *G*_1 _into *G*_2 _with strictly less than *δ *events, a contradiction. Hence, we assume that only one of *β*_1 _and *β*_2 _is created by *σ*_*i*_ and without loss of generality, let *β*_1 _= *α*_1_. Now, we consider the following two cases.

Case 1: *σ*_*i*+1 _is a fusion. For simplicity of discussion, we let *α *= (1, 2,..., *x *- 1, *x*,..., *y *- 1), *β*_2 _= (*y*, *y *+ 1,..., *z*), *σ*_*i *_= (1, *x*) and *σ*_*i*+1 _= (1, *y*), where 1 <*x *<*y *- 1 and *y *<*z*. Then the net rearrangement caused by *σ*_*i*_ and *σ*_*i*+1 _is to transform *α *and *β *into *α*_2 _= (*x*,*x*+1,...,*y *- 1) and *β *= (1, 2,..., *x *- 1, *y*, *y *+ 1,..., *z*). In fact, this rearrangement can also be done by first joining *α *and *β*_2 _into (1, 2,..., *z*) via *σ*_*i*+1 _and then splitting (1, 2,..., *z*) into *α*_2 _and *β *via (*x*, *y*). In other words, we can obtain Φ' by letting σ′i
 MathType@MTEF@5@5@+=feaafiart1ev1aaatCvAUfKttLearuWrP9MDH5MBPbIqV92AaeXatLxBI9gBaebbnrfifHhDYfgasaacH8akY=wiFfYdH8Gipec8Eeeu0xXdbba9frFj0=OqFfea0dXdd9vqai=hGuQ8kuc9pgc9s8qqaq=dirpe0xb9q8qiLsFr0=vr0=vr0dc8meaabaqaciaacaGaaeqabaqabeGadaaakeaaiiGacuWFdpWCgaqbamaaBaaaleaaieGacqGFPbqAaeqaaaaa@300F@ = *σ*_*i*+1 _and σ′i+1
 MathType@MTEF@5@5@+=feaafiart1ev1aaatCvAUfeBSjuyZL2yd9gzLbvyNv2CaerbwvMCKfMBHbqedmvETj2BSbqee0evGueE0jxyaibaieIgFLIOYR2NHOxjYhrPYhrPYpI8F4rqqrFfpeea0xe9Lq=Jc9vqaqpepm0xbbG8FasPYRqj0=yi0lXdbba9pGe9qqFf0dXdHuk9fr=xfr=xfrpiWZqaaeaabiGaaiaacaqabeaabeqacmaaaOqaaGGaciqb=n8aZzaafaWaaSbaaSqaamXvP5wqonvsaeHbhL2B2fMBULgic92BRbaceiGae4xAaKgceaGae03kaSIae0xmaedabeaaaaa@4216@ = (*x*, *y*).

Case 2: *σ*_*i*+1 _is a block-interchange. Clearly, the net rearrangement caused by *σ*_*i*_ and *σ*_*i*+1 _is to transform *α *into *β *and *α*_2_, which is equivalent to the rearrangement by first applying *σ*_*i*+1_to *α *and then further splitting it into *β *and *α*_2 _via *σ*_*i*_. Then Φ' is obtained by swapping *σ*_*i*_ and *σ*_*i*+1 _in Φ.

In other words, we can always obtain Φ' from Φ according to the method described above. Repeating this process on the resulting Φ', we can finally obtain an optimal series of events that are required to transform *G*_1 _into *G*_2 _such that all fissions come after all fusions and block-interchanges.

**Lemma 4 ***There is an optimal series of events required to transform G*_1 _*into G*_2 _*in a canonical order such that all fusions come before all block-interchanges, which come before all fissions*.

*Proof*. Let Φ = <*σ*_1_, *σ*_2_,..., *σ*_*δ*_> be an optimal series of events required to transform *G*_1 _into *G*_2_. If there are no fusions or block-interchanges, then the proof is completed. If not, according to Lemma 3, we may assume that all fusions and block-interchanges occur earlier than all fissions. Let *i *be the index of the last non-fission in Φ and also let *G*' be the resulting genome after all *σ*_1_, *σ*_2_,...*σ*_*i *_have affected *G*_1_. Since Φ is optimal, it is straightforward to see that Φ_*i *_= <*σ*_1_, *σ*_2_,...,*σ*_*i*_> is an optimal series of fusions and block-interchanges needed to transform *G*_1 _into *G*'. As discussed in the proof of Lemma 2, Φ′i
 MathType@MTEF@5@5@+=feaafiart1ev1aaatCvAUfKttLearuWrP9MDH5MBPbIqV92AaeXatLxBI9gBaebbnrfifHhDYfgasaacH8akY=wiFfYdH8Gipec8Eeeu0xXdbba9frFj0=OqFfea0dXdd9vqai=hGuQ8kuc9pgc9s8qqaq=dirpe0xb9q8qiLsFr0=vr0=vr0dc8meaabaqaciaacaGaaeqabaqabeGadaaakeaacuqHMoGrgaqbamaaBaaaleaacqWGPbqAaeqaaaaa@2FB9@ = <*σ*_*i*_, *σ*_*i*-1_,...,*σ*_1_> is an optimal series of fissions and block-interchanges for transforming *G*' into *G*_1_. Moreover, by Lemma 3, we can obtain Φ′′i
 MathType@MTEF@5@5@+=feaafiart1ev1aaatCvAUfKttLearuWrP9MDH5MBPbIqV92AaeXatLxBI9gBaebbnrfifHhDYfgasaacH8akY=wiFfYdH8Gipec8Eeeu0xXdbba9frFj0=OqFfea0dXdd9vqai=hGuQ8kuc9pgc9s8qqaq=dirpe0xb9q8qiLsFr0=vr0=vr0dc8meaabaqaciaacaGaaeqabaqabeGadaaakeaacuqHMoGrgaqbgaqbamaaBaaaleaacqWGPbqAaeqaaaaa@2FC4@ = <σ′i
 MathType@MTEF@5@5@+=feaafiart1ev1aaatCvAUfKttLearuWrP9MDH5MBPbIqV92AaeXatLxBI9gBaebbnrfifHhDYfgasaacH8akY=wiFfYdH8Gipec8Eeeu0xXdbba9frFj0=OqFfea0dXdd9vqai=hGuQ8kuc9pgc9s8qqaq=dirpe0xb9q8qiLsFr0=vr0=vr0dc8meaabaqaciaacaGaaeqabaqabeGadaaakeaaiiGacuWFdpWCgaqbamaaBaaaleaaieGacqGFPbqAaeqaaaaa@300F@, σ′i−1
 MathType@MTEF@5@5@+=feaafiart1ev1aaatCvAUfKttLearuWrP9MDH5MBPbIqV92AaeXatLxBI9gBaebbnrfifHhDYfgasaacH8akY=wiFfYdH8Gipec8Eeeu0xXdbba9frFj0=OqFfea0dXdd9vqai=hGuQ8kuc9pgc9s8qqaq=dirpe0xb9q8qiLsFr0=vr0=vr0dc8meaabaqaciaacaGaaeqabaqabeGadaaakeaaiiGacuWFdpWCgaqbamaaBaaaleaacqWGPbqAcqGHsislcqaIXaqmaeqaaaaa@31E6@,...,σ′1
 MathType@MTEF@5@5@+=feaafiart1ev1aaatCvAUfKttLearuWrP9MDH5MBPbIqV92AaeXatLxBI9gBaebbnrfifHhDYfgasaacH8akY=wiFfYdH8Gipec8Eeeu0xXdbba9frFj0=OqFfea0dXdd9vqai=hGuQ8kuc9pgc9s8qqaq=dirpe0xb9q8qiLsFr0=vr0=vr0dc8meaabaqaciaacaGaaeqabaqabeGadaaakeaaiiGacuWFdpWCgaqbamaaBaaaleaaiiaacqGFXaqmaeqaaaaa@2F9E@> from Φ′i
 MathType@MTEF@5@5@+=feaafiart1ev1aaatCvAUfKttLearuWrP9MDH5MBPbIqV92AaeXatLxBI9gBaebbnrfifHhDYfgasaacH8akY=wiFfYdH8Gipec8Eeeu0xXdbba9frFj0=OqFfea0dXdd9vqai=hGuQ8kuc9pgc9s8qqaq=dirpe0xb9q8qiLsFr0=vr0=vr0dc8meaabaqaciaacaGaaeqabaqabeGadaaakeaacuqHMoGrgaqbamaaBaaaleaacqWGPbqAaeqaaaaa@2FB9@ for transforming *G*' into *G*_1 _such that all block- interchanges in Φ′′i
 MathType@MTEF@5@5@+=feaafiart1ev1aaatCvAUfKttLearuWrP9MDH5MBPbIqV92AaeXatLxBI9gBaebbnrfifHhDYfgasaacH8akY=wiFfYdH8Gipec8Eeeu0xXdbba9frFj0=OqFfea0dXdd9vqai=hGuQ8kuc9pgc9s8qqaq=dirpe0xb9q8qiLsFr0=vr0=vr0dc8meaabaqaciaacaGaaeqabaqabeGadaaakeaacuqHMoGrgaqbgaqbamaaBaaaleaacqWGPbqAaeqaaaaa@2FC4@ occur prior to all fissions. Consequently, <σ′1
 MathType@MTEF@5@5@+=feaafiart1ev1aaatCvAUfKttLearuWrP9MDH5MBPbIqV92AaeXatLxBI9gBaebbnrfifHhDYfgasaacH8akY=wiFfYdH8Gipec8Eeeu0xXdbba9frFj0=OqFfea0dXdd9vqai=hGuQ8kuc9pgc9s8qqaq=dirpe0xb9q8qiLsFr0=vr0=vr0dc8meaabaqaciaacaGaaeqabaqabeGadaaakeaaiiGacuWFdpWCgaqbamaaBaaaleaaiiaacqGFXaqmaeqaaaaa@2F9E@, σ′2
 MathType@MTEF@5@5@+=feaafiart1ev1aaatCvAUfKttLearuWrP9MDH5MBPbIqV92AaeXatLxBI9gBaebbnrfifHhDYfgasaacH8akY=wiFfYdH8Gipec8Eeeu0xXdbba9frFj0=OqFfea0dXdd9vqai=hGuQ8kuc9pgc9s8qqaq=dirpe0xb9q8qiLsFr0=vr0=vr0dc8meaabaqaciaacaGaaeqabaqabeGadaaakeaaiiGacuWFdpWCgaqbamaaBaaaleaacqaIYaGmaeqaaaaa@2FA0@,...,σ′i
 MathType@MTEF@5@5@+=feaafiart1ev1aaatCvAUfKttLearuWrP9MDH5MBPbIqV92AaeXatLxBI9gBaebbnrfifHhDYfgasaacH8akY=wiFfYdH8Gipec8Eeeu0xXdbba9frFj0=OqFfea0dXdd9vqai=hGuQ8kuc9pgc9s8qqaq=dirpe0xb9q8qiLsFr0=vr0=vr0dc8meaabaqaciaacaGaaeqabaqabeGadaaakeaaiiGacuWFdpWCgaqbamaaBaaaleaaieGacqGFPbqAaeqaaaaa@300F@> is an optimal series of fusions and block-interchanges needed to transform *G*_1 _into *G*', and all its fusions occur before all its block-interchanges. Therefore, there is an optimal series of events needed to transform *G*_1 _into *G*_2 _such that all fusions come earlier than all block-interchanges, which come before all fissions.     □

It is worth mentioning that an optimal scenario in a canonical order does not necessarily exist for linear multi-chromosomal genomes. For example, suppose that *G*_1 _and *G*_2 _are two given linear multi-chromosomal genomes, where *G*_1 _= (1, 4, 5) (2, 3) and *G*_2 _= (1, 2, 3) (4, 5). Then the optimal scenario between them is a fission, splitting (1, 4, 5) into (1) (4, 5), followed by a fusion, joining (1) and (2, 3) to (1, 2, 3). However, this optimal scenario can not be transformed into another in the canonical order according to the steps as described in Lemmas 3 and 4. Actually, there is no an optimal scenario between such two linear genomes using any two rearrangement events that begin with a fusion.

#### Algorithm

Let *α *and *I *be two given circular multi-chromosomal genomes over the same gene set *E *= {1, 2,...,*n*}. Here, we assume that the genes in *I *are sorted in the order of increasing and consecutive numbers, and that gene *i *+ 1 is on the right side of gene *i *within the same chromosome, where 1 ≤ *i *≤ *n *- 1. For example, *I *= (1, 2) (3, 4, 5) (6, 7, 8, 9) if *I *has three circular chromosomes with two, three and four genes, respectively. In this case, the computation of *d*(*α*,*I*) and its corresponding optimal scenario can be considered as a problem of *sorting α *using the minimum set of operations, including fusions, fissions and block-interchanges.

Suppose that *ρ*_*λ*_*ρ*_*λ *- 1 _... *ρ*_1 _is a product of 2-cycles that corresponds to an optimal series Φ of fusions, fissions and block-interchanges for transforming *α *into *I*. Then Φ = *ρ*_*λ*_*ρ*_*λ *- 1_...*ρ*_1 _= *Iα*^-1 ^and by Lemma 1, *λ *≥ *n *- *f*(*Iα*^-1^). If Φ' = ρ′λρ′λ−1…ρ′1
 MathType@MTEF@5@5@+=feaafiart1ev1aaatCvAUfKttLearuWrP9MDH5MBPbIqV92AaeXatLxBI9gBaebbnrfifHhDYfgasaacH8akY=wiFfYdH8Gipec8Eeeu0xXdbba9frFj0=OqFfea0dXdd9vqai=hGuQ8kuc9pgc9s8qqaq=dirpe0xb9q8qiLsFr0=vr0=vr0dc8meaabaqaciaacaGaaeqabaqabeGadaaakeaaiiGacuWFbpGCgaqbamaaBaaaleaacqWF7oaBaeqaaOGaf8xWdiNbauaadaWgaaWcbaGae83UdWMaeyOeI0IaeGymaedabeaakiablAciljqb=f8aYzaafaWaaSbaaSqaaiabigdaXaqabaaaaa@39F2@ is another product of *λ *2-cycles with Φ' = *Iα*^-1^, then the number of 2-cycles in Φ' that function as fusions or fissions must be greater than or equal to that in Φ; otherwise, the total number of fusions, fissions and block-interchanges in Φ' for transforming *α *into *I *must be less than that in Φ, a contradiction. The reason is that a fusion or fission requires only one 2-cycle for rearrangement, whereas a block-interchange requires two 2-cycles. In other words, the number of 2-cycles serving as the fusions and fissions is minimum in any optimal series of events.

Based on the above observation as well as Lemma 4, below we design an efficient algorithm for computing *d*(*α*,*I*) and its optimal scenario of rearrangement events in a canonical order. Let *χ*(*α*) and *χ*(*I*) denote the numbers of chromosomes in *α *and *I*, respectively, and let *α *= *α*_1_*α*_2_...*α*_*χ*(*α*) _and *I *= *I*_1_*I*_2_...*I*_*χ*(*I*)_. Then, an undirected graph G
 MathType@MTEF@5@5@+=feaafiart1ev1aaatCvAUfKttLearuWrP9MDH5MBPbIqV92AaeXatLxBI9gBamrtHrhAL1wy0L2yHvtyaeHbnfgDOvwBHrxAJfwnaebbnrfifHhDYfgasaacH8akY=wiFfYdH8Gipec8Eeeu0xXdbba9frFj0=OqFfea0dXdd9vqai=hGuQ8kuc9pgc9s8qqaq=dirpe0xb9q8qiLsFr0=vr0=vr0dc8meaabaqaciaacaGaaeqabaWaaeGaeaaakeaaimaacqWFge=raaa@382D@(*α*,*I*) = (V
 MathType@MTEF@5@5@+=feaafiart1ev1aaatCvAUfKttLearuWrP9MDH5MBPbIqV92AaeXatLxBI9gBamrtHrhAL1wy0L2yHvtyaeHbnfgDOvwBHrxAJfwnaebbnrfifHhDYfgasaacH8akY=wiFfYdH8Gipec8Eeeu0xXdbba9frFj0=OqFfea0dXdd9vqai=hGuQ8kuc9pgc9s8qqaq=dirpe0xb9q8qiLsFr0=vr0=vr0dc8meaabaqaciaacaGaaeqabaWaaeGaeaaakeaaimaacqWFveVvaaa@384B@_*α*_, V
 MathType@MTEF@5@5@+=feaafiart1ev1aaatCvAUfKttLearuWrP9MDH5MBPbIqV92AaeXatLxBI9gBamrtHrhAL1wy0L2yHvtyaeHbnfgDOvwBHrxAJfwnaebbnrfifHhDYfgasaacH8akY=wiFfYdH8Gipec8Eeeu0xXdbba9frFj0=OqFfea0dXdd9vqai=hGuQ8kuc9pgc9s8qqaq=dirpe0xb9q8qiLsFr0=vr0=vr0dc8meaabaqaciaacaGaaeqabaWaaeGaeaaakeaaimaacqWFveVvaaa@384B@_*I*_, ℰ
 MathType@MTEF@5@5@+=feaafiart1ev1aaatCvAUfKttLearuWrP9MDH5MBPbIqV92AaeXatLxBI9gBamrtHrhAL1wy0L2yHvtyaeHbnfgDOvwBHrxAJfwnaebbnrfifHhDYfgasaacH8akY=wiFfYdH8Gipec8Eeeu0xXdbba9frFj0=OqFfea0dXdd9vqai=hGuQ8kuc9pgc9s8qqaq=dirpe0xb9q8qiLsFr0=vr0=vr0dc8meaabaqaciaacaGaaeqabaWaaeGaeaaakeaaimaacqWFWesraaa@3785@) is constructed from *α *and *I *as follows.

• V
 MathType@MTEF@5@5@+=feaafiart1ev1aaatCvAUfKttLearuWrP9MDH5MBPbIqV92AaeXatLxBI9gBamrtHrhAL1wy0L2yHvtyaeHbnfgDOvwBHrxAJfwnaebbnrfifHhDYfgasaacH8akY=wiFfYdH8Gipec8Eeeu0xXdbba9frFj0=OqFfea0dXdd9vqai=hGuQ8kuc9pgc9s8qqaq=dirpe0xb9q8qiLsFr0=vr0=vr0dc8meaabaqaciaacaGaaeqabaWaaeGaeaaakeaaimaacqWFveVvaaa@384B@_*α *_= {*α*_1_, *α*_2_,...,*α*_*χ*(*α*)_}.

• V
 MathType@MTEF@5@5@+=feaafiart1ev1aaatCvAUfKttLearuWrP9MDH5MBPbIqV92AaeXatLxBI9gBamrtHrhAL1wy0L2yHvtyaeHbnfgDOvwBHrxAJfwnaebbnrfifHhDYfgasaacH8akY=wiFfYdH8Gipec8Eeeu0xXdbba9frFj0=OqFfea0dXdd9vqai=hGuQ8kuc9pgc9s8qqaq=dirpe0xb9q8qiLsFr0=vr0=vr0dc8meaabaqaciaacaGaaeqabaWaaeGaeaaakeaaimaacqWFveVvaaa@384B@_*I *_= {*I*_1_, *I*_2_,...,*I*_*χ*(*I*)_}.

• ℰ
 MathType@MTEF@5@5@+=feaafiart1ev1aaatCvAUfKttLearuWrP9MDH5MBPbIqV92AaeXatLxBI9gBamrtHrhAL1wy0L2yHvtyaeHbnfgDOvwBHrxAJfwnaebbnrfifHhDYfgasaacH8akY=wiFfYdH8Gipec8Eeeu0xXdbba9frFj0=OqFfea0dXdd9vqai=hGuQ8kuc9pgc9s8qqaq=dirpe0xb9q8qiLsFr0=vr0=vr0dc8meaabaqaciaacaGaaeqabaWaaeGaeaaakeaaimaacqWFWesraaa@3785@ = {(*α*_*i*_, *I*_*j*_) | 1 ≤ *i *≤ *χ *(*α*), 1 ≤ *j *≤ *χ *(*I*), and *α*_*i *_and *I*_*j *_have at least a common gene}.

**Figure 2:**
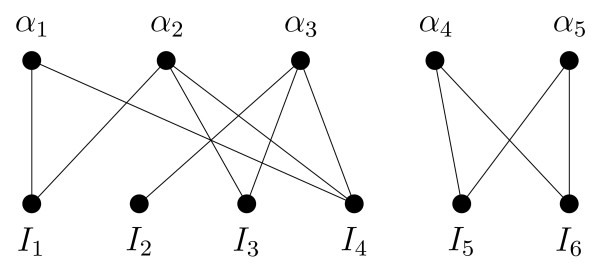
The induced bipartite graph G
 MathType@MTEF@5@5@+=feaafiart1ev1aaatCvAUfKttLearuWrP9MDH5MBPbIqV92AaeXatLxBI9gBamrtHrhAL1wy0L2yHvtyaeHbnfgDOvwBHrxAJfwnaebbnrfifHhDYfgasaacH8akY=wiFfYdH8Gipec8Eeeu0xXdbba9frFj0=OqFfea0dXdd9vqai=hGuQ8kuc9pgc9s8qqaq=dirpe0xb9q8qiLsFr0=vr0=vr0dc8meaabaqaciaacaGaaeqabaWaaeGaeaaakeaaimaacqWFge=raaa@382D@(*α*, *I*) with two connected components.

For instance, suppose that *α *= *α*_1_*α*_2_...*α*_5 _= (1, 2, 10) (11, 8, 9, 3, 6) (7, 4, 5,12) (13, 15) (14, 16) and *I *= *I*_1_*I*_2 _... *I*_6 _= (1, 2, 3) (4, 5) (6, 7, 8) (9, 10, 11, 12) (13, 14) (15, 16). Then the induced graph G
 MathType@MTEF@5@5@+=feaafiart1ev1aaatCvAUfKttLearuWrP9MDH5MBPbIqV92AaeXatLxBI9gBamrtHrhAL1wy0L2yHvtyaeHbnfgDOvwBHrxAJfwnaebbnrfifHhDYfgasaacH8akY=wiFfYdH8Gipec8Eeeu0xXdbba9frFj0=OqFfea0dXdd9vqai=hGuQ8kuc9pgc9s8qqaq=dirpe0xb9q8qiLsFr0=vr0=vr0dc8meaabaqaciaacaGaaeqabaWaaeGaeaaakeaaimaacqWFge=raaa@382D@(*α*,*I*) is shown in Figure 2, which is a bipartite graph since V
 MathType@MTEF@5@5@+=feaafiart1ev1aaatCvAUfKttLearuWrP9MDH5MBPbIqV92AaeXatLxBI9gBamrtHrhAL1wy0L2yHvtyaeHbnfgDOvwBHrxAJfwnaebbnrfifHhDYfgasaacH8akY=wiFfYdH8Gipec8Eeeu0xXdbba9frFj0=OqFfea0dXdd9vqai=hGuQ8kuc9pgc9s8qqaq=dirpe0xb9q8qiLsFr0=vr0=vr0dc8meaabaqaciaacaGaaeqabaWaaeGaeaaakeaaimaacqWFveVvaaa@384B@_*α *_and V
 MathType@MTEF@5@5@+=feaafiart1ev1aaatCvAUfKttLearuWrP9MDH5MBPbIqV92AaeXatLxBI9gBamrtHrhAL1wy0L2yHvtyaeHbnfgDOvwBHrxAJfwnaebbnrfifHhDYfgasaacH8akY=wiFfYdH8Gipec8Eeeu0xXdbba9frFj0=OqFfea0dXdd9vqai=hGuQ8kuc9pgc9s8qqaq=dirpe0xb9q8qiLsFr0=vr0=vr0dc8meaabaqaciaacaGaaeqabaWaaeGaeaaakeaaimaacqWFveVvaaa@384B@_*I *_are independent sets in G
 MathType@MTEF@5@5@+=feaafiart1ev1aaatCvAUfKttLearuWrP9MDH5MBPbIqV92AaeXatLxBI9gBamrtHrhAL1wy0L2yHvtyaeHbnfgDOvwBHrxAJfwnaebbnrfifHhDYfgasaacH8akY=wiFfYdH8Gipec8Eeeu0xXdbba9frFj0=OqFfea0dXdd9vqai=hGuQ8kuc9pgc9s8qqaq=dirpe0xb9q8qiLsFr0=vr0=vr0dc8meaabaqaciaacaGaaeqabaWaaeGaeaaakeaaimaacqWFge=raaa@382D@(*α*, *I*) (i.e., no edge between any two vertices in V
 MathType@MTEF@5@5@+=feaafiart1ev1aaatCvAUfKttLearuWrP9MDH5MBPbIqV92AaeXatLxBI9gBamrtHrhAL1wy0L2yHvtyaeHbnfgDOvwBHrxAJfwnaebbnrfifHhDYfgasaacH8akY=wiFfYdH8Gipec8Eeeu0xXdbba9frFj0=OqFfea0dXdd9vqai=hGuQ8kuc9pgc9s8qqaq=dirpe0xb9q8qiLsFr0=vr0=vr0dc8meaabaqaciaacaGaaeqabaWaaeGaeaaakeaaimaacqWFveVvaaa@384B@_*α *_or V
 MathType@MTEF@5@5@+=feaafiart1ev1aaatCvAUfKttLearuWrP9MDH5MBPbIqV92AaeXatLxBI9gBamrtHrhAL1wy0L2yHvtyaeHbnfgDOvwBHrxAJfwnaebbnrfifHhDYfgasaacH8akY=wiFfYdH8Gipec8Eeeu0xXdbba9frFj0=OqFfea0dXdd9vqai=hGuQ8kuc9pgc9s8qqaq=dirpe0xb9q8qiLsFr0=vr0=vr0dc8meaabaqaciaacaGaaeqabaWaaeGaeaaakeaaimaacqWFveVvaaa@384B@_*I*_). A *connected component *of G
 MathType@MTEF@5@5@+=feaafiart1ev1aaatCvAUfKttLearuWrP9MDH5MBPbIqV92AaeXatLxBI9gBamrtHrhAL1wy0L2yHvtyaeHbnfgDOvwBHrxAJfwnaebbnrfifHhDYfgasaacH8akY=wiFfYdH8Gipec8Eeeu0xXdbba9frFj0=OqFfea0dXdd9vqai=hGuQ8kuc9pgc9s8qqaq=dirpe0xb9q8qiLsFr0=vr0=vr0dc8meaabaqaciaacaGaaeqabaWaaeGaeaaakeaaimaacqWFge=raaa@382D@(*α*,*I*) is defined to be a maximal subgraph of G
 MathType@MTEF@5@5@+=feaafiart1ev1aaatCvAUfKttLearuWrP9MDH5MBPbIqV92AaeXatLxBI9gBamrtHrhAL1wy0L2yHvtyaeHbnfgDOvwBHrxAJfwnaebbnrfifHhDYfgasaacH8akY=wiFfYdH8Gipec8Eeeu0xXdbba9frFj0=OqFfea0dXdd9vqai=hGuQ8kuc9pgc9s8qqaq=dirpe0xb9q8qiLsFr0=vr0=vr0dc8meaabaqaciaacaGaaeqabaWaaeGaeaaakeaaimaacqWFge=raaa@382D@(*α*,*I*) such that there exists a path between any pair of vertices in this subgraph. For example, the induced G
 MathType@MTEF@5@5@+=feaafiart1ev1aaatCvAUfKttLearuWrP9MDH5MBPbIqV92AaeXatLxBI9gBamrtHrhAL1wy0L2yHvtyaeHbnfgDOvwBHrxAJfwnaebbnrfifHhDYfgasaacH8akY=wiFfYdH8Gipec8Eeeu0xXdbba9frFj0=OqFfea0dXdd9vqai=hGuQ8kuc9pgc9s8qqaq=dirpe0xb9q8qiLsFr0=vr0=vr0dc8meaabaqaciaacaGaaeqabaWaaeGaeaaakeaaimaacqWFge=raaa@382D@(*α*,*I*) as shown in Figure 2 has two connected components. Notice that if in a chromosome *I*_*k *_of *I *there are two genes that appear in two different chromosomes *α*_*i*_ and *α*_*j *_of *α*, then (*α*_*i*_, *I*_*k*_) ∈ ℰ
 MathType@MTEF@5@5@+=feaafiart1ev1aaatCvAUfKttLearuWrP9MDH5MBPbIqV92AaeXatLxBI9gBamrtHrhAL1wy0L2yHvtyaeHbnfgDOvwBHrxAJfwnaebbnrfifHhDYfgasaacH8akY=wiFfYdH8Gipec8Eeeu0xXdbba9frFj0=OqFfea0dXdd9vqai=hGuQ8kuc9pgc9s8qqaq=dirpe0xb9q8qiLsFr0=vr0=vr0dc8meaabaqaciaacaGaaeqabaWaaeGaeaaakeaaimaacqWFWesraaa@3785@ and (*α*_*j*_, *I*_*k*_) ∈ ℰ
 MathType@MTEF@5@5@+=feaafiart1ev1aaatCvAUfKttLearuWrP9MDH5MBPbIqV92AaeXatLxBI9gBamrtHrhAL1wy0L2yHvtyaeHbnfgDOvwBHrxAJfwnaebbnrfifHhDYfgasaacH8akY=wiFfYdH8Gipec8Eeeu0xXdbba9frFj0=OqFfea0dXdd9vqai=hGuQ8kuc9pgc9s8qqaq=dirpe0xb9q8qiLsFr0=vr0=vr0dc8meaabaqaciaacaGaaeqabaWaaeGaeaaakeaaimaacqWFWesraaa@3785@, and hence both *α*_*i*_ and *α*_*j*_ belong to the same connected component in G
 MathType@MTEF@5@5@+=feaafiart1ev1aaatCvAUfKttLearuWrP9MDH5MBPbIqV92AaeXatLxBI9gBamrtHrhAL1wy0L2yHvtyaeHbnfgDOvwBHrxAJfwnaebbnrfifHhDYfgasaacH8akY=wiFfYdH8Gipec8Eeeu0xXdbba9frFj0=OqFfea0dXdd9vqai=hGuQ8kuc9pgc9s8qqaq=dirpe0xb9q8qiLsFr0=vr0=vr0dc8meaabaqaciaacaGaaeqabaWaaeGaeaaakeaaimaacqWFge=raaa@382D@(*α*,*I*).

Let {C
 MathType@MTEF@5@5@+=feaafiart1ev1aaatCvAUfKttLearuWrP9MDH5MBPbIqV92AaeXatLxBI9gBamrtHrhAL1wy0L2yHvtyaeHbnfgDOvwBHrxAJfwnaebbnrfifHhDYfgasaacH8akY=wiFfYdH8Gipec8Eeeu0xXdbba9frFj0=OqFfea0dXdd9vqai=hGuQ8kuc9pgc9s8qqaq=dirpe0xb9q8qiLsFr0=vr0=vr0dc8meaabaqaciaacaGaaeqabaWaaeGaeaaakeaaimaacqWFce=qaaa@3825@_1_, C
 MathType@MTEF@5@5@+=feaafiart1ev1aaatCvAUfKttLearuWrP9MDH5MBPbIqV92AaeXatLxBI9gBamrtHrhAL1wy0L2yHvtyaeHbnfgDOvwBHrxAJfwnaebbnrfifHhDYfgasaacH8akY=wiFfYdH8Gipec8Eeeu0xXdbba9frFj0=OqFfea0dXdd9vqai=hGuQ8kuc9pgc9s8qqaq=dirpe0xb9q8qiLsFr0=vr0=vr0dc8meaabaqaciaacaGaaeqabaWaaeGaeaaakeaaimaacqWFce=qaaa@3825@_2_,...,C
 MathType@MTEF@5@5@+=feaafiart1ev1aaatCvAUfKttLearuWrP9MDH5MBPbIqV92AaeXatLxBI9gBamrtHrhAL1wy0L2yHvtyaeHbnfgDOvwBHrxAJfwnaebbnrfifHhDYfgasaacH8akY=wiFfYdH8Gipec8Eeeu0xXdbba9frFj0=OqFfea0dXdd9vqai=hGuQ8kuc9pgc9s8qqaq=dirpe0xb9q8qiLsFr0=vr0=vr0dc8meaabaqaciaacaGaaeqabaWaaeGaeaaakeaaimaacqWFce=qaaa@3825@_*ω*_} denote the collection of all connected components in G
 MathType@MTEF@5@5@+=feaafiart1ev1aaatCvAUfKttLearuWrP9MDH5MBPbIqV92AaeXatLxBI9gBamrtHrhAL1wy0L2yHvtyaeHbnfgDOvwBHrxAJfwnaebbnrfifHhDYfgasaacH8akY=wiFfYdH8Gipec8Eeeu0xXdbba9frFj0=OqFfea0dXdd9vqai=hGuQ8kuc9pgc9s8qqaq=dirpe0xb9q8qiLsFr0=vr0=vr0dc8meaabaqaciaacaGaaeqabaWaaeGaeaaakeaaimaacqWFge=raaa@382D@(*α*,*I*). For each 1 ≤ *i *≤ *ω*, let *β*_*i *_and *J*_*i*_ denote the chromosomes in *α *and *I*, respectively, whose corresponding vertices belong to C
 MathType@MTEF@5@5@+=feaafiart1ev1aaatCvAUfKttLearuWrP9MDH5MBPbIqV92AaeXatLxBI9gBamrtHrhAL1wy0L2yHvtyaeHbnfgDOvwBHrxAJfwnaebbnrfifHhDYfgasaacH8akY=wiFfYdH8Gipec8Eeeu0xXdbba9frFj0=OqFfea0dXdd9vqai=hGuQ8kuc9pgc9s8qqaq=dirpe0xb9q8qiLsFr0=vr0=vr0dc8meaabaqaciaacaGaaeqabaWaaeGaeaaakeaaimaacqWFce=qaaa@3825@_*i *_in G
 MathType@MTEF@5@5@+=feaafiart1ev1aaatCvAUfKttLearuWrP9MDH5MBPbIqV92AaeXatLxBI9gBamrtHrhAL1wy0L2yHvtyaeHbnfgDOvwBHrxAJfwnaebbnrfifHhDYfgasaacH8akY=wiFfYdH8Gipec8Eeeu0xXdbba9frFj0=OqFfea0dXdd9vqai=hGuQ8kuc9pgc9s8qqaq=dirpe0xb9q8qiLsFr0=vr0=vr0dc8meaabaqaciaacaGaaeqabaWaaeGaeaaakeaaimaacqWFge=raaa@382D@(*α*,*I*). Let gene(*β*_*i*_) and gene(*J*_*i*_) be the collections of the genes in all chromosomes of *β*_*i *_and *J*_*i*_, respectively. Then gene(*β*_*i*_) = gene(*J*_*i*_) and gene(*β*_*i*_) ∩ gene(*β*_*j*_) = ∅ for any 1 ≤ *j *≠ *i *≤ *ω*. Let *n*_*i*_ be the number of genes in gene(*β*_*i*_). Clearly, *n *= *n*_1 _+ *n*_2 _+ ... + *n*_*ω*_. In addition, it can be verified that *Iα*^-1 ^= (*J*_1_β1−1
 MathType@MTEF@5@5@+=feaafiart1ev1aaatCvAUfKttLearuWrP9MDH5MBPbIqV92AaeXatLxBI9gBaebbnrfifHhDYfgasaacH8akY=wiFfYdH8Gipec8Eeeu0xXdbba9frFj0=OqFfea0dXdd9vqai=hGuQ8kuc9pgc9s8qqaq=dirpe0xb9q8qiLsFr0=vr0=vr0dc8meaabaqaciaacaGaaeqabaqabeGadaaakeaaiiGacqWFYoGydaqhaaWcbaGaeGymaedabaGaeyOeI0IaeGymaedaaaaa@314E@) (*J*_2_β2−1
 MathType@MTEF@5@5@+=feaafiart1ev1aaatCvAUfKttLearuWrP9MDH5MBPbIqV92AaeXatLxBI9gBaebbnrfifHhDYfgasaacH8akY=wiFfYdH8Gipec8Eeeu0xXdbba9frFj0=OqFfea0dXdd9vqai=hGuQ8kuc9pgc9s8qqaq=dirpe0xb9q8qiLsFr0=vr0=vr0dc8meaabaqaciaacaGaaeqabaqabeGadaaakeaaiiGacqWFYoGydaqhaaWcbaGaeGOmaidabaGaeyOeI0IaeGymaedaaaaa@3150@) ... (*J*_*ω*_βω−1
 MathType@MTEF@5@5@+=feaafiart1ev1aaatCvAUfKttLearuWrP9MDH5MBPbIqV92AaeXatLxBI9gBaebbnrfifHhDYfgasaacH8akY=wiFfYdH8Gipec8Eeeu0xXdbba9frFj0=OqFfea0dXdd9vqai=hGuQ8kuc9pgc9s8qqaq=dirpe0xb9q8qiLsFr0=vr0=vr0dc8meaabaqaciaacaGaaeqabaqabeGadaaakeaaiiGacqWFYoGydaqhaaWcbaGae8xYdChabaGaeyOeI0IaeGymaedaaaaa@3226@) and *f*(*Iα*^-1^) = *f*(*J*_1_β1−1
 MathType@MTEF@5@5@+=feaafiart1ev1aaatCvAUfKttLearuWrP9MDH5MBPbIqV92AaeXatLxBI9gBaebbnrfifHhDYfgasaacH8akY=wiFfYdH8Gipec8Eeeu0xXdbba9frFj0=OqFfea0dXdd9vqai=hGuQ8kuc9pgc9s8qqaq=dirpe0xb9q8qiLsFr0=vr0=vr0dc8meaabaqaciaacaGaaeqabaqabeGadaaakeaaiiGacqWFYoGydaqhaaWcbaGaeGymaedabaGaeyOeI0IaeGymaedaaaaa@314E@) + *f*(*J*_2_β2−1
 MathType@MTEF@5@5@+=feaafiart1ev1aaatCvAUfKttLearuWrP9MDH5MBPbIqV92AaeXatLxBI9gBaebbnrfifHhDYfgasaacH8akY=wiFfYdH8Gipec8Eeeu0xXdbba9frFj0=OqFfea0dXdd9vqai=hGuQ8kuc9pgc9s8qqaq=dirpe0xb9q8qiLsFr0=vr0=vr0dc8meaabaqaciaacaGaaeqabaqabeGadaaakeaaiiGacqWFYoGydaqhaaWcbaGaeGOmaidabaGaeyOeI0IaeGymaedaaaaa@3150@) + ... + *f*(*J*_*ω*_βω−1
 MathType@MTEF@5@5@+=feaafiart1ev1aaatCvAUfKttLearuWrP9MDH5MBPbIqV92AaeXatLxBI9gBaebbnrfifHhDYfgasaacH8akY=wiFfYdH8Gipec8Eeeu0xXdbba9frFj0=OqFfea0dXdd9vqai=hGuQ8kuc9pgc9s8qqaq=dirpe0xb9q8qiLsFr0=vr0=vr0dc8meaabaqaciaacaGaaeqabaqabeGadaaakeaaiiGacqWFYoGydaqhaaWcbaGae8xYdChabaGaeyOeI0IaeGymaedaaaaa@3226@).

According to the properties above, we then find a product Φ of 2-cycles so that Φ*α *= *I *as follows. We first find a product Φ_*i *_of 2-cycles that corresponds to an optimal series of the rearrangement events required to transform *β*_*i *_into *J*_*i *_(i.e., Φ_*i*_*β*_*i *_= *J*_*i*_ and hence Φ_*i *_= *J*_*i*_βj−1
 MathType@MTEF@5@5@+=feaafiart1ev1aaatCvAUfKttLearuWrP9MDH5MBPbIqV92AaeXatLxBI9gBaebbnrfifHhDYfgasaacH8akY=wiFfYdH8Gipec8Eeeu0xXdbba9frFj0=OqFfea0dXdd9vqai=hGuQ8kuc9pgc9s8qqaq=dirpe0xb9q8qiLsFr0=vr0=vr0dc8meaabaqaciaacaGaaeqabaqabeGadaaakeaaiiGacqWFYoGydaqhaaWcbaGaemOAaOgabaGaeyOeI0IaeGymaedaaaaa@31BB@) and then let Φ = Φ_*ω*_Φ_*ω*-1 _... Φ_1_. Clearly, Φ = *Iα*^-1 ^and hence Φ corresponds to a feasible series of events for transforming *α *into *I*. Actually, we shall show later that the number of 2-cycles in each Φ_*i *_is *n*_*i *_- *f*(*J*_*i*_βi−1
 MathType@MTEF@5@5@+=feaafiart1ev1aaatCvAUfKttLearuWrP9MDH5MBPbIqV92AaeXatLxBI9gBaebbnrfifHhDYfgasaacH8akY=wiFfYdH8Gipec8Eeeu0xXdbba9frFj0=OqFfea0dXdd9vqai=hGuQ8kuc9pgc9s8qqaq=dirpe0xb9q8qiLsFr0=vr0=vr0dc8meaabaqaciaacaGaaeqabaqabeGadaaakeaaiiGacqWFYoGydaqhaaWcbaGaemyAaKgabaGaeyOeI0IaeGymaedaaaaa@31B9@), and in Φ_*i*_ the number of 2-cycles functioning as the fusions and fissions is minimum. This causes that the number of 2-cycles in Φ equals to ∑i=1ωni−f(Jiβi−1)=n−f(Iα−1)
 MathType@MTEF@5@5@+=feaafiart1ev1aaatCvAUfKttLearuWrP9MDH5MBPbIqV92AaeXatLxBI9gBaebbnrfifHhDYfgasaacH8akY=wiFfYdH8Gipec8Eeeu0xXdbba9frFj0=OqFfea0dXdd9vqai=hGuQ8kuc9pgc9s8qqaq=dirpe0xb9q8qiLsFr0=vr0=vr0dc8meaabaqaciaacaGaaeqabaqabeGadaaakeaadaaeWaqaaiabd6gaUnaaBaaaleaacqWGPbqAaeqaaaqaaiabdMgaPjabg2da9iabigdaXaqaaGGaciab=L8a3bqdcqGHris5aOGaeyOeI0IaemOzayMaeiikaGIaemOsaO0aaSbaaSqaaiabdMgaPbqabaGccqWFYoGydaqhaaWcbaGaemyAaKgabaGaeyOeI0IaeGymaedaaOGaeiykaKIaeyypa0JaemOBa4MaeyOeI0IaemOzayMaeiikaGIaemysaKKae8xSde2aaWbaaSqabeaacqGHsislcqaIXaqmaaGccqGGPaqkaaa@4D93@, in which the number of 2-cycles serving as the fusions and fissions is minimum. As a result, Φ is an optimal series of events for transforming *α *into *I*. The above description indicates that the original problem can be conquered by independently solving the same problem on the smaller instance whose induced bipartite graph is a connected component of G
 MathType@MTEF@5@5@+=feaafiart1ev1aaatCvAUfKttLearuWrP9MDH5MBPbIqV92AaeXatLxBI9gBamrtHrhAL1wy0L2yHvtyaeHbnfgDOvwBHrxAJfwnaebbnrfifHhDYfgasaacH8akY=wiFfYdH8Gipec8Eeeu0xXdbba9frFj0=OqFfea0dXdd9vqai=hGuQ8kuc9pgc9s8qqaq=dirpe0xb9q8qiLsFr0=vr0=vr0dc8meaabaqaciaacaGaaeqabaWaaeGaeaaakeaaimaacqWFge=raaa@382D@(*α*, *I*).

To simplify our discussion, throughout the rest of this section we assume that the induced G
 MathType@MTEF@5@5@+=feaafiart1ev1aaatCvAUfKttLearuWrP9MDH5MBPbIqV92AaeXatLxBI9gBamrtHrhAL1wy0L2yHvtyaeHbnfgDOvwBHrxAJfwnaebbnrfifHhDYfgasaacH8akY=wiFfYdH8Gipec8Eeeu0xXdbba9frFj0=OqFfea0dXdd9vqai=hGuQ8kuc9pgc9s8qqaq=dirpe0xb9q8qiLsFr0=vr0=vr0dc8meaabaqaciaacaGaaeqabaWaaeGaeaaakeaaimaacqWFge=raaa@382D@(*α*, *I*) of a given instance *α *and *I *has exactly one connected component. Let Φ = <*σ*_1_, *σ*_2_, ...., *σ*_*δ*_> be an optimal series of events for transforming *α *into *I *in which all fusions precede all block-interchanges that further precede all fissions. Let *n*_*fu*_, *n*_*bi *_and *n*_*fi *_denote the numbers of fusions, block-interchanges and fissions, respectively, in Φ. Then *δ *= *n*_*fu *_+ *n*_*bi *_+ *n*_*fi*_. In the following, we shall show that Φ can be expressed by a product of *n *- *f*(*Iα*^-1^) 2-cycles in which the number of 2-cycles functioning as the fusions and fissions is minimum.

It should be noticed that the chromosomes considered here are disjoint (i.e., without gene duplication). Hence, for any two chromosomes *α*_*i*_ and *α*_*j *_in *α *with (*α*_*i*_*I*_*k*_) ∈ ℰ
 MathType@MTEF@5@5@+=feaafiart1ev1aaatCvAUfKttLearuWrP9MDH5MBPbIqV92AaeXatLxBI9gBamrtHrhAL1wy0L2yHvtyaeHbnfgDOvwBHrxAJfwnaebbnrfifHhDYfgasaacH8akY=wiFfYdH8Gipec8Eeeu0xXdbba9frFj0=OqFfea0dXdd9vqai=hGuQ8kuc9pgc9s8qqaq=dirpe0xb9q8qiLsFr0=vr0=vr0dc8meaabaqaciaacaGaaeqabaWaaeGaeaaakeaaimaacqWFWesraaa@3785@ and (*α*_*j*_, *I*_*k*_) ∈ ℰ
 MathType@MTEF@5@5@+=feaafiart1ev1aaatCvAUfKttLearuWrP9MDH5MBPbIqV92AaeXatLxBI9gBamrtHrhAL1wy0L2yHvtyaeHbnfgDOvwBHrxAJfwnaebbnrfifHhDYfgasaacH8akY=wiFfYdH8Gipec8Eeeu0xXdbba9frFj0=OqFfea0dXdd9vqai=hGuQ8kuc9pgc9s8qqaq=dirpe0xb9q8qiLsFr0=vr0=vr0dc8meaabaqaciaacaGaaeqabaWaaeGaeaaakeaaimaacqWFWesraaa@3785@, there must exist a fusion in Φ that joins *α*_*i*_ and *α*_*j *_to one chromosome; otherwise, *I*_*k *_can not be formed from *α *by a fission later. Since all needed fusions come together in the beginning of Φ, *n*_*fu *_= *χ*(*α*) - 1, which is the lower bound of the number of fusions required in any optimal series of events for transforming *α *into *I*. After these *n*_*fu *_fusions, the resulting *α *becomes only one chromosome. Since the next *n*_*bi *_block-interchanges are intra-chromosomal mutations, we have *n*_*fi *_= *χ*(*I*) - 1. Actually, *χ*(*I*) - 1 is the minimum number of the required fissions in any optimal series of events for transforming *α *into *I*, since it is the minimum number of the fusions used in the corresponding optimal series of events to transform *I *into *α*.

Given any cycle *ρ*, we use *x *∈ *ρ *to denote that *x *is a number in *ρ*. For any two *x *∈ *ρ *and *y *∈ *ρ*, they are said to be *adjacent *in *ρ *if *ρ*(*x*) = *y *or *ρ*(*y*) = *x*. Next, we show a way to derive *n*_*fu *_2-cycles from *Iα*^-1 ^such that these 2-cycles function as the fusions that join all chromosomes of *α *to a single one, if *α *has multiple chromosomes, where *n*_*fu *_= *χ*(*α*) - 1. For simplicity, later in the text we use "cycle in *Iα*^-1^" to represent " cycle in the cycle decomposition of *Iα*^-1^" in meaning, unless a possible confusion may arise.

**Lemma 5 ***Let α*_*i *_*and α*_*j *_*be any two disjoint cycles in α*. *Then there must exist a cycle in Iα*^-1 ^*that contains two numbers x and y such that x *∈ *α*_*i *_*and y *∈ *α*_*j*_.

*Proof*. Since we assume that the induced G
 MathType@MTEF@5@5@+=feaafiart1ev1aaatCvAUfKttLearuWrP9MDH5MBPbIqV92AaeXatLxBI9gBamrtHrhAL1wy0L2yHvtyaeHbnfgDOvwBHrxAJfwnaebbnrfifHhDYfgasaacH8akY=wiFfYdH8Gipec8Eeeu0xXdbba9frFj0=OqFfea0dXdd9vqai=hGuQ8kuc9pgc9s8qqaq=dirpe0xb9q8qiLsFr0=vr0=vr0dc8meaabaqaciaacaGaaeqabaWaaeGaeaaakeaaimaacqWFge=raaa@382D@(*α*, *I*) contains exactly and only one connected component, and *α*_*i *_and *α*_*j *_contain some numbers *u *and *v*, respectively, such that both *u *and *v *are in a cycle *I*_*k *_of *I*. Notice that *u *∉ *α*_*j *_and *v *∉ *α*_*i*_. Suppose that there is no cycle in *Iα*^-1 ^that contains two numbers *x *and *y *such that they are in these two different cycles of *α*, say *x *∈ *α*_*i*_ and *y *∈ *α*_*j*_. Then all numbers in any cycle of *Iα*^-1 ^are contained in some cycle of *α*. Without loss of generality, let *I*_*k *_= (*u *≡ *a*_1_, *a*_2_, ..., *a*_*p *_≡ *v*, ..., *a*_*q*_) and let *p *<*q *for simplifying the discussion. For each 1 ≤ *x *≤ *p*, let *α*(*a*_*x*_) = *b*_*x*_. Then we have *Iα*^-1^(*b*_*x*_) = *a*_*x*+1 _(since *Iα*^-1^*α *= *I*), which means that both *b*_*x *_and *a*_*x*+1 _are in the same cycle of *Iα*^-1 ^and hence they are also in the same cycle of *α*. If *a*_*x *_is in *α*_*i*_, then *b*_*x *_is also in *α*_*i*_, which further leads to *a*_*x*+1 _∈ *α*_*i*_. Since *u *= *a*_1 _is in *α*_*i*_, all *a*_2_, *a*_3_, ..., *a*_*p *_are in *α*_*i*_. As a result, both of *u *and *v *are in *α*_*i*_, a contradiction. Hence, there exists a cycle in *Iα*^-1 ^that contains *x *and *y *such that *x *∈ *α*_*i *_and *y *∈ *α*_*j*_.

The following lemma can be easily verified.

**Lemma 6 **(*a*_1_, *a*_2_,..., *a*_*i*_,..., *a*_*j*_) = (*a*_1_, *a*_2_,..., *a*_*i*_) (*a*_*i*+1_,..., *a*_*j*_) (*a*_*i*_, *a*_*j*_), *where *1 ≤ *i *<*j*.

According to Lemma 5, for any two cycles *α*_*i*_ and *α*_*j *_of *α*, we can find two numbers *x *and *y *in a cycle of *Iα*^-1^, say *β*, such that *x *∈ *α*_*i*_ and *y *∈ *α*_*j*_. Let *β *= (*a*_1_, *a*_2_,..., *a*_*q*_), where *q *≥ 2. Then we consider the following two cases. Case 1: *x *and *y *are adjacent in *β*. For simplicity, let *x *= *a*_*q*-1 _and *y *= *a*_*q*_. Then by Lemma 6, *β *= (*a*_1_, *a*_2_,..., *a*_*q*-1_) (*a*_*q*_) (*x*, *y*). Case 2: *x *and *y *are not adjacent in *β*. Let *x *= *a*_*p *_and *y *= *a*_*q*_, where 1 ≤ *p *<*q *- 1. Then *β *= (*a*_1_, *a*_2_,..., *a*_*p*_) (*a*_*p*+1_,..., *a*_*q*_) (*x*,*y*) according to Lemma 6. In other words, we can derive a 2-cycle (*x*, *y*) from *β *such that it can join *α*_*i*_ and *α*_*j *_to one cycle. After *α*_*i*_, and *α*_*j *_are joined together via (*x*, *y*), the number of the cycles (including 1-cycles) in the resulting *Iα*^-1 ^increases by one. Repeatedly based on the procedure above, we can derive consecutive *n*_*fu *_2-cycles from *Iα*^-1^, say *φ*_1_, *φ*_2_,...,φnfu
 MathType@MTEF@5@5@+=feaafiart1ev1aaatCvAUfKttLearuWrP9MDH5MBPbIqV92AaeXatLxBI9gBaebbnrfifHhDYfgasaacH8akY=wiFfYdH8Gipec8Eeeu0xXdbba9frFj0=OqFfea0dXdd9vqai=hGuQ8kuc9pgc9s8qqaq=dirpe0xb9q8qiLsFr0=vr0=vr0dc8meaabaqaciaacaGaaeqabaqabeGadaaakeaaiiGacqWFgpGzdaWgaaWcbaGaemOBa42aaSbaaWqaaiabdAgaMjabdwha1bqabaaaleqaaaaa@32FD@, that can join *χ *(*α*) cycles in *α *to a single one, where *n*_*fu *_= *χ*(*α*) - 1. In other words, *φ*_1_, *φ*_2_,...,φnfu
 MathType@MTEF@5@5@+=feaafiart1ev1aaatCvAUfKttLearuWrP9MDH5MBPbIqV92AaeXatLxBI9gBaebbnrfifHhDYfgasaacH8akY=wiFfYdH8Gipec8Eeeu0xXdbba9frFj0=OqFfea0dXdd9vqai=hGuQ8kuc9pgc9s8qqaq=dirpe0xb9q8qiLsFr0=vr0=vr0dc8meaabaqaciaacaGaaeqabaqabeGadaaakeaaiiGacqWFgpGzdaWgaaWcbaGaemOBa42aaSbaaWqaaiabdAgaMjabdwha1bqabaaaleqaaaaa@32FD@ function as *χ*(*α*) - 1 fusions that transform genome *α *with *χ*(*α*) chromosomes into a genome, denoted by *α*', with a single chromosome. Clearly, we have *α*' = φnfu
 MathType@MTEF@5@5@+=feaafiart1ev1aaatCvAUfKttLearuWrP9MDH5MBPbIqV92AaeXatLxBI9gBaebbnrfifHhDYfgasaacH8akY=wiFfYdH8Gipec8Eeeu0xXdbba9frFj0=OqFfea0dXdd9vqai=hGuQ8kuc9pgc9s8qqaq=dirpe0xb9q8qiLsFr0=vr0=vr0dc8meaabaqaciaacaGaaeqabaqabeGadaaakeaaiiGacqWFgpGzdaWgaaWcbaGaemOBa42aaSbaaWqaaiabdAgaMjabdwha1bqabaaaleqaaaaa@32FD@φnfu−1
 MathType@MTEF@5@5@+=feaafiart1ev1aaatCvAUfKttLearuWrP9MDH5MBPbIqV92AaeXatLxBI9gBaebbnrfifHhDYfgasaacH8akY=wiFfYdH8Gipec8Eeeu0xXdbba9frFj0=OqFfea0dXdd9vqai=hGuQ8kuc9pgc9s8qqaq=dirpe0xb9q8qiLsFr0=vr0=vr0dc8meaabaqaciaacaGaaeqabaqabeGadaaakeaaiiGacqWFgpGzdaWgaaWcbaGaemOBa42aaSbaaWqaaiabdAgaMjabdwha1bqabaWccqGHsislcqaIXaqmaeqaaaaa@34DA@...*φ*_1_*α*, *Iα*'^-1 ^= *Iα*^-1 ^*φ*_1_*φ*_2 _... φnfu
 MathType@MTEF@5@5@+=feaafiart1ev1aaatCvAUfKttLearuWrP9MDH5MBPbIqV92AaeXatLxBI9gBaebbnrfifHhDYfgasaacH8akY=wiFfYdH8Gipec8Eeeu0xXdbba9frFj0=OqFfea0dXdd9vqai=hGuQ8kuc9pgc9s8qqaq=dirpe0xb9q8qiLsFr0=vr0=vr0dc8meaabaqaciaacaGaaeqabaqabeGadaaakeaaiiGacqWFgpGzdaWgaaWcbaGaemOBa42aaSbaaWqaaiabdAgaMjabdwha1bqabaaaleqaaaaa@32FD@, and *f*(*Iα*'^-1^) = *f*(*Iα*^-1^) + *n*_*fu*_. Hence, we can immediately claim the following.

**Claim 1 ***α*' = φnfu
 MathType@MTEF@5@5@+=feaafiart1ev1aaatCvAUfKttLearuWrP9MDH5MBPbIqV92AaeXatLxBI9gBaebbnrfifHhDYfgasaacH8akY=wiFfYdH8Gipec8Eeeu0xXdbba9frFj0=OqFfea0dXdd9vqai=hGuQ8kuc9pgc9s8qqaq=dirpe0xb9q8qiLsFr0=vr0=vr0dc8meaabaqaciaacaGaaeqabaqabeGadaaakeaaiiGacqWFgpGzdaWgaaWcbaGaemOBa42aaSbaaWqaaiabdAgaMjabdwha1bqabaaaleqaaaaa@32FD@φnfu−1
 MathType@MTEF@5@5@+=feaafiart1ev1aaatCvAUfKttLearuWrP9MDH5MBPbIqV92AaeXatLxBI9gBaebbnrfifHhDYfgasaacH8akY=wiFfYdH8Gipec8Eeeu0xXdbba9frFj0=OqFfea0dXdd9vqai=hGuQ8kuc9pgc9s8qqaq=dirpe0xb9q8qiLsFr0=vr0=vr0dc8meaabaqaciaacaGaaeqabaqabeGadaaakeaaiiGacqWFgpGzdaWgaaWcbaGaemOBa42aaSbaaWqaaiabdAgaMjabdwha1bqabaWccqGHsislcqaIXaqmaeqaaaaa@34DA@...*φ*_1_*α*, *Iα*'^-1 ^= *Iα*^-1 ^*φ*_1_*φ*_2 _... φnfu
 MathType@MTEF@5@5@+=feaafiart1ev1aaatCvAUfKttLearuWrP9MDH5MBPbIqV92AaeXatLxBI9gBaebbnrfifHhDYfgasaacH8akY=wiFfYdH8Gipec8Eeeu0xXdbba9frFj0=OqFfea0dXdd9vqai=hGuQ8kuc9pgc9s8qqaq=dirpe0xb9q8qiLsFr0=vr0=vr0dc8meaabaqaciaacaGaaeqabaqabeGadaaakeaaiiGacqWFgpGzdaWgaaWcbaGaemOBa42aaSbaaWqaaiabdAgaMjabdwha1bqabaaaleqaaaaa@32FD@, and *f*(*Iα*'^-1^) = *f*(*Iα*^-1^) + *n*_*fu*_, where *n*_*fu *_= *χ*(*α*) - 1.

Without loss of generality, we now suppose that *χ*(*I*) > 1. Similarly as the discussion above, we can derive consecutive *n*_*fi *_2-cycles from *α*'*I*^-1^, say *ψ*_1_, *ψ*_2_, ..., ψnfi
 MathType@MTEF@5@5@+=feaafiart1ev1aaatCvAUfKttLearuWrP9MDH5MBPbIqV92AaeXatLxBI9gBaebbnrfifHhDYfgasaacH8akY=wiFfYdH8Gipec8Eeeu0xXdbba9frFj0=OqFfea0dXdd9vqai=hGuQ8kuc9pgc9s8qqaq=dirpe0xb9q8qiLsFr0=vr0=vr0dc8meaabaqaciaacaGaaeqabaqabeGadaaakeaaiiGacqWFipqEdaWgaaWcbaGaemOBa42aaSbaaWqaaiabdAgaMjabdMgaPbqabaaaleqaaaaa@32FA@, such that they serve as the fusions to transform *I *with *χ*(*I*) chromosomes into a genome, denoted by *I*', with only one chromosome, where *n*_*fi *_= *χ*(*I*) - 1 and *α*'*I*^-1 ^is the inverse of *Iα*'^-1 ^(i.e., *α*'*I*^-1 ^= (*Iα*'^-1^)^-1^). Then we have *I*' = ψnfi
 MathType@MTEF@5@5@+=feaafiart1ev1aaatCvAUfKttLearuWrP9MDH5MBPbIqV92AaeXatLxBI9gBaebbnrfifHhDYfgasaacH8akY=wiFfYdH8Gipec8Eeeu0xXdbba9frFj0=OqFfea0dXdd9vqai=hGuQ8kuc9pgc9s8qqaq=dirpe0xb9q8qiLsFr0=vr0=vr0dc8meaabaqaciaacaGaaeqabaqabeGadaaakeaaiiGacqWFipqEdaWgaaWcbaGaemOBa42aaSbaaWqaaiabdAgaMjabdMgaPbqabaaaleqaaaaa@32FA@ψnfi−1
 MathType@MTEF@5@5@+=feaafiart1ev1aaatCvAUfKttLearuWrP9MDH5MBPbIqV92AaeXatLxBI9gBaebbnrfifHhDYfgasaacH8akY=wiFfYdH8Gipec8Eeeu0xXdbba9frFj0=OqFfea0dXdd9vqai=hGuQ8kuc9pgc9s8qqaq=dirpe0xb9q8qiLsFr0=vr0=vr0dc8meaabaqaciaacaGaaeqabaqabeGadaaakeaaiiGacqWFipqEdaWgaaWcbaGaemOBa42aaSbaaWqaaiabdAgaMjabdMgaPbqabaWccqGHsislcqaIXaqmaeqaaaaa@34D7@ ... *ψ*_1_*I *(hence *ψ*_1_*ψ*_2 _... ψnfi
 MathType@MTEF@5@5@+=feaafiart1ev1aaatCvAUfKttLearuWrP9MDH5MBPbIqV92AaeXatLxBI9gBaebbnrfifHhDYfgasaacH8akY=wiFfYdH8Gipec8Eeeu0xXdbba9frFj0=OqFfea0dXdd9vqai=hGuQ8kuc9pgc9s8qqaq=dirpe0xb9q8qiLsFr0=vr0=vr0dc8meaabaqaciaacaGaaeqabaqabeGadaaakeaaiiGacqWFipqEdaWgaaWcbaGaemOBa42aaSbaaWqaaiabdAgaMjabdMgaPbqabaaaleqaaaaa@32FA@*I*' = *I*), *α*'*I*'^-1 ^= *α*'*I*^-1^*ψ*_1_*ψ*_2 _... ψnfi
 MathType@MTEF@5@5@+=feaafiart1ev1aaatCvAUfKttLearuWrP9MDH5MBPbIqV92AaeXatLxBI9gBaebbnrfifHhDYfgasaacH8akY=wiFfYdH8Gipec8Eeeu0xXdbba9frFj0=OqFfea0dXdd9vqai=hGuQ8kuc9pgc9s8qqaq=dirpe0xb9q8qiLsFr0=vr0=vr0dc8meaabaqaciaacaGaaeqabaqabeGadaaakeaaiiGacqWFipqEdaWgaaWcbaGaemOBa42aaSbaaWqaaiabdAgaMjabdMgaPbqabaaaleqaaaaa@32FA@ and *f*(*α*'*I*'^-1^) = *f*(*α*'*I*^-1^) + *n*_*fi*_. Conversely, we can use ψnfi
 MathType@MTEF@5@5@+=feaafiart1ev1aaatCvAUfKttLearuWrP9MDH5MBPbIqV92AaeXatLxBI9gBaebbnrfifHhDYfgasaacH8akY=wiFfYdH8Gipec8Eeeu0xXdbba9frFj0=OqFfea0dXdd9vqai=hGuQ8kuc9pgc9s8qqaq=dirpe0xb9q8qiLsFr0=vr0=vr0dc8meaabaqaciaacaGaaeqabaqabeGadaaakeaaiiGacqWFipqEdaWgaaWcbaGaemOBa42aaSbaaWqaaiabdAgaMjabdMgaPbqabaaaleqaaaaa@32FA@, ψnfi−1
 MathType@MTEF@5@5@+=feaafiart1ev1aaatCvAUfKttLearuWrP9MDH5MBPbIqV92AaeXatLxBI9gBaebbnrfifHhDYfgasaacH8akY=wiFfYdH8Gipec8Eeeu0xXdbba9frFj0=OqFfea0dXdd9vqai=hGuQ8kuc9pgc9s8qqaq=dirpe0xb9q8qiLsFr0=vr0=vr0dc8meaabaqaciaacaGaaeqabaqabeGadaaakeaaiiGacqWFipqEdaWgaaWcbaGaemOBa42aaSbaaWqaaiabdAgaMjabdMgaPbqabaWccqGHsislcqaIXaqmaeqaaaaa@34D7@…, *ψ*_1 _as fissions to split *I*' with one chromosome into *I *with *n*_*fi *_chromosomes. Since *α*'*I*^-1 ^is the inverse of *Iα*'^-1^, it can be easily obtained from *Iα*'^-1 ^by just reversing the order of the numbers in each cycle of *Iα*'^-1 ^and hence *f*(*α*'*I*^-1^) = *f*(*Iα*'^-1^), which leads to *f*(*α*'*I*'^-1^) = *f*(*Iα*'^-1^) + *n*_*fi*_. As a result, we have *f*(*I*'*α*'^-1^) = *f*(*α*'*I*^-1^) = *f*(*Iα*'^-1^) + *n*_*fi *_since *I*'*α*'^-1 ^= (*α*'*I*^-1^)^-1^. Therefore, the following claim can be obtained.

**Claim 2 ***ψ*_1_*ψ*_2 _... ψnfi
 MathType@MTEF@5@5@+=feaafiart1ev1aaatCvAUfKttLearuWrP9MDH5MBPbIqV92AaeXatLxBI9gBaebbnrfifHhDYfgasaacH8akY=wiFfYdH8Gipec8Eeeu0xXdbba9frFj0=OqFfea0dXdd9vqai=hGuQ8kuc9pgc9s8qqaq=dirpe0xb9q8qiLsFr0=vr0=vr0dc8meaabaqaciaacaGaaeqabaqabeGadaaakeaaiiGacqWFipqEdaWgaaWcbaGaemOBa42aaSbaaWqaaiabdAgaMjabdMgaPbqabaaaleqaaaaa@32FA@*I*' = *I*, *α*'*I*'^-1 ^= *α*'*I*^-1^*ψ*_1_*ψ*_2 _... ψnfi
 MathType@MTEF@5@5@+=feaafiart1ev1aaatCvAUfKttLearuWrP9MDH5MBPbIqV92AaeXatLxBI9gBaebbnrfifHhDYfgasaacH8akY=wiFfYdH8Gipec8Eeeu0xXdbba9frFj0=OqFfea0dXdd9vqai=hGuQ8kuc9pgc9s8qqaq=dirpe0xb9q8qiLsFr0=vr0=vr0dc8meaabaqaciaacaGaaeqabaqabeGadaaakeaaiiGacqWFipqEdaWgaaWcbaGaemOBa42aaSbaaWqaaiabdAgaMjabdMgaPbqabaaaleqaaaaa@32FA@*and f*(*I*'*α*'^-1^) = *f*(*Iα*'^-1^) + *n*_*fi*_, *where n*_*fi *_= *χ*(*I*) - 1.

Notice that both *α*' and *I*' now are the genomes with only one chromosome. Then based on the algorithm proposed by Lin *et al*. [14], we can find nbi=n−f(I′α′−1)2
 MathType@MTEF@5@5@+=feaafiart1ev1aaatCvAUfKttLearuWrP9MDH5MBPbIqV92AaeXatLxBI9gBaebbnrfifHhDYfgasaacH8akY=wiFfYdH8Gipec8Eeeu0xXdbba9frFj0=OqFfea0dXdd9vqai=hGuQ8kuc9pgc9s8qqaq=dirpe0xb9q8qiLsFr0=vr0=vr0dc8meaabaqaciaacaGaaeqabaqabeGadaaakeaacqWGUbGBdaWgaaWcbaGaemOyaiMaemyAaKgabeaakiabg2da9maalaaabaGaemOBa4MaeyOeI0IaemOzayMaeiikaGIafmysaKKbauaaiiGacuWFXoqygaqbamaaCaaaleqabaaccaGae4NeI0Iae4xmaedaaOGaeiykaKcabaGaeGOmaidaaaaa@3D35@ block-interchanges from *I*'*α*'^-1 ^to transform *α*' into *I*'. Certainly, these *n*_*bi *_block-interchanges can be further expressed by a product of 2*n*_*bi *_2-cycles, say τ11,τ12,τ21,τ22,…,τnbi1,τnbi2
 MathType@MTEF@5@5@+=feaafiart1ev1aaatCvAUfKttLearuWrP9MDH5MBPbIqV92AaeXatLxBI9gBaebbnrfifHhDYfgasaacH8akY=wiFfYdH8Gipec8Eeeu0xXdbba9frFj0=OqFfea0dXdd9vqai=hGuQ8kuc9pgc9s8qqaq=dirpe0xb9q8qiLsFr0=vr0=vr0dc8meaabaqaciaacaGaaeqabaqabeGadaaakeaaiiGacqWFepaDdaqhaaWcbaGaeGymaedabaGaeGymaedaaOGaeiilaWIae8hXdq3aa0baaSqaaiabigdaXaqaaiabikdaYaaakiabcYcaSiab=r8a0naaDaaaleaacqaIYaGmaeaacqaIXaqmaaGccqGGSaalcqWFepaDdaqhaaWcbaGaeGOmaidabaGaeGOmaidaaOGaeiilaWIaeSOjGSKaeiilaWIae8hXdq3aa0baaSqaaiabd6gaUnaaBaaameaacqWGIbGycqWGPbqAaeqaaaWcbaGaeGymaedaaOGaeiilaWIae8hXdq3aa0baaSqaaiabd6gaUnaaBaaameaacqWGIbGycqWGPbqAaeqaaaWcbaGaeGOmaidaaaaa@50CE@, such that every two consecutive 2-cycles act as a block-interchange in the process of transforming *α*' into *I*', where *I*'*α*'^-1 ^= τnbi2τnbi1τnbi−12τnbi−11…τ12τ11
 MathType@MTEF@5@5@+=feaafiart1ev1aaatCvAUfKttLearuWrP9MDH5MBPbIqV92AaeXatLxBI9gBaebbnrfifHhDYfgasaacH8akY=wiFfYdH8Gipec8Eeeu0xXdbba9frFj0=OqFfea0dXdd9vqai=hGuQ8kuc9pgc9s8qqaq=dirpe0xb9q8qiLsFr0=vr0=vr0dc8meaabaqaciaacaGaaeqabaqabeGadaaakeaaiiGacqWFepaDdaqhaaWcbaGaemOBa42aaSbaaWqaaiabdkgaIjabdMgaPbqabaaaleaacqaIYaGmaaGccqWFepaDdaqhaaWcbaGaemOBa42aaSbaaWqaaiabdkgaIjabdMgaPbqabaaaleaacqaIXaqmaaGccqWFepaDdaqhaaWcbaGaemOBa42aaSbaaWqaaiabdkgaIjabdMgaPbqabaWccqGHsislcqaIXaqmaeaacqaIYaGmaaGccqWFepaDdaqhaaWcbaGaemOBa42aaSbaaWqaaiabdkgaIjabdMgaPbqabaWccqGHsislcqaIXaqmaeaacqaIXaqmaaGccqWIMaYscqWFepaDdaqhaaWcbaGaeGymaedabaGaeGOmaidaaOGae8hXdq3aa0baaSqaaiabigdaXaqaaiabigdaXaaaaaa@55EE@. Hence, we have the following claim immediately.

**Claim 3 ***I' *= τnbi2τnbi1τnbi−12τnbi−11…τ12τ11
 MathType@MTEF@5@5@+=feaafiart1ev1aaatCvAUfKttLearuWrP9MDH5MBPbIqV92AaeXatLxBI9gBaebbnrfifHhDYfgasaacH8akY=wiFfYdH8Gipec8Eeeu0xXdbba9frFj0=OqFfea0dXdd9vqai=hGuQ8kuc9pgc9s8qqaq=dirpe0xb9q8qiLsFr0=vr0=vr0dc8meaabaqaciaacaGaaeqabaqabeGadaaakeaaiiGacqWFepaDdaqhaaWcbaGaemOBa42aaSbaaWqaaiabdkgaIjabdMgaPbqabaaaleaacqaIYaGmaaGccqWFepaDdaqhaaWcbaGaemOBa42aaSbaaWqaaiabdkgaIjabdMgaPbqabaaaleaacqaIXaqmaaGccqWFepaDdaqhaaWcbaGaemOBa42aaSbaaWqaaiabdkgaIjabdMgaPbqabaWccqGHsislcqaIXaqmaeaacqaIYaGmaaGccqWFepaDdaqhaaWcbaGaemOBa42aaSbaaWqaaiabdkgaIjabdMgaPbqabaWccqGHsislcqaIXaqmaeaacqaIXaqmaaGccqWIMaYscqWFepaDdaqhaaWcbaGaeGymaedabaGaeGOmaidaaOGae8hXdq3aa0baaSqaaiabigdaXaqaaiabigdaXaaaaaa@55EE@*α'*.

Now we let Φ = *ψ*_1_*ψ*_2_...ψnfiτnbi2τnbi1…τ12τ11φnfuφnfu−1…φ1
 MathType@MTEF@5@5@+=feaafiart1ev1aaatCvAUfKttLearuWrP9MDH5MBPbIqV92AaeXatLxBI9gBaebbnrfifHhDYfgasaacH8akY=wiFfYdH8Gipec8Eeeu0xXdbba9frFj0=OqFfea0dXdd9vqai=hGuQ8kuc9pgc9s8qqaq=dirpe0xb9q8qiLsFr0=vr0=vr0dc8meaabaqaciaacaGaaeqabaqabeGadaaakeaaiiGacqWFipqEdaWgaaWcbaGaemOBa42aaSbaaWqaaiabdAgaMjabdMgaPbqabaaaleqaaOGae8hXdq3aa0baaSqaaiabd6gaUnaaBaaameaacqWGIbGycqWGPbqAaeqaaaWcbaGaeGOmaidaaOGae8hXdq3aa0baaSqaaiabd6gaUnaaBaaameaacqWGIbGycqWGPbqAaeqaaaWcbaGaeGymaedaaOGaeSOjGSKae8hXdq3aa0baaSqaaiabigdaXaqaaiabikdaYaaakiab=r8a0naaDaaaleaacqaIXaqmaeaacqaIXaqmaaGccqWFgpGzdaWgaaWcbaGaemOBa42aaSbaaWqaaiabdAgaMjabdwha1bqabaaaleqaaOGae8NXdy2aaSbaaSqaaiabd6gaUnaaBaaameaacqWGMbGzcqWG1bqDaeqaaSGaeyOeI0IaeGymaedabeaakiablAciljab=z8aMnaaBaaaleaacqaIXaqmaeqaaaaa@5C9D@. Then the result of Φ*α *= *I *(hence Φ = *Iα*^-1^) can be easily verified by Claims 1, 2 and 3 as follows.



In other words, Φ is a product of (*n*_*fu *_+ (*n *- *f*(*I*'*α*'^-1^)) + *n*_*fi*_) 2-cycles that can transform *α *into *I*. More clearly, Φ first uses *φ*_1_, *φ*_2_,...,φnfu
 MathType@MTEF@5@5@+=feaafiart1ev1aaatCvAUfKttLearuWrP9MDH5MBPbIqV92AaeXatLxBI9gBaebbnrfifHhDYfgasaacH8akY=wiFfYdH8Gipec8Eeeu0xXdbba9frFj0=OqFfea0dXdd9vqai=hGuQ8kuc9pgc9s8qqaq=dirpe0xb9q8qiLsFr0=vr0=vr0dc8meaabaqaciaacaGaaeqabaqabeGadaaakeaaiiGacqWFgpGzdaWgaaWcbaGaemOBa42aaSbaaWqaaiabdAgaMjabdwha1bqabaaaleqaaaaa@32FD@ (acting as *n*_*fu *_fusions) to transform *α *into *α*', then uses τ11,τ12,…,τnbi1,τnbi2
 MathType@MTEF@5@5@+=feaafiart1ev1aaatCvAUfKttLearuWrP9MDH5MBPbIqV92AaeXatLxBI9gBaebbnrfifHhDYfgasaacH8akY=wiFfYdH8Gipec8Eeeu0xXdbba9frFj0=OqFfea0dXdd9vqai=hGuQ8kuc9pgc9s8qqaq=dirpe0xb9q8qiLsFr0=vr0=vr0dc8meaabaqaciaacaGaaeqabaqabeGadaaakeaaiiGacqWFepaDdaqhaaWcbaGaeGymaedabaGaeGymaedaaOGaeiilaWIae8hXdq3aa0baaSqaaiabigdaXaqaaiabikdaYaaakiabcYcaSiablAciljabcYcaSiab=r8a0naaDaaaleaacqWGUbGBdaWgaaadbaGaemOyaiMaemyAaKgabeaaaSqaaiabigdaXaaakiabcYcaSiab=r8a0naaDaaaleaacqWGUbGBdaWgaaadbaGaemOyaiMaemyAaKgabeaaaSqaaiabikdaYaaaaaa@475A@ (acting as *n*_*bi *_block-interchanges) to transform *α*' into *I*', and finally uses ψnfi
 MathType@MTEF@5@5@+=feaafiart1ev1aaatCvAUfKttLearuWrP9MDH5MBPbIqV92AaeXatLxBI9gBaebbnrfifHhDYfgasaacH8akY=wiFfYdH8Gipec8Eeeu0xXdbba9frFj0=OqFfea0dXdd9vqai=hGuQ8kuc9pgc9s8qqaq=dirpe0xb9q8qiLsFr0=vr0=vr0dc8meaabaqaciaacaGaaeqabaqabeGadaaakeaaiiGacqWFipqEdaWgaaWcbaGaemOBa42aaSbaaWqaaiabdAgaMjabdMgaPbqabaaaleqaaaaa@32FA@, ψnfi−1
 MathType@MTEF@5@5@+=feaafiart1ev1aaatCvAUfKttLearuWrP9MDH5MBPbIqV92AaeXatLxBI9gBaebbnrfifHhDYfgasaacH8akY=wiFfYdH8Gipec8Eeeu0xXdbba9frFj0=OqFfea0dXdd9vqai=hGuQ8kuc9pgc9s8qqaq=dirpe0xb9q8qiLsFr0=vr0=vr0dc8meaabaqaciaacaGaaeqabaqabeGadaaakeaaiiGacqWFipqEdaWgaaWcbaGaemOBa42aaSbaaWqaaiabdAgaMjabdMgaPbqabaWccqGHsislcqaIXaqmaeqaaaaa@34D7@,..., *ψ*_1 _(acting as *n*_*fi *_fissions) to transform *I*' into *I*. By Claims 1 and 2, we can show that (*n*_*fu *_+ (*n *- *f*(*I*'*α*'^-1^)) + *n*_*fi *_= *n *- *f*(*Iα*^-1^) as follows.

*n*_*fu *_+ (*n *- *f*(*I*'*α*'^-1^)) + *n*_*fi*_

= *n*_*fu *_+ (*n *- *f*(*Iα*'^-1^) + *n*_*fi*_) + *n*_*fi *_    (by Claim 2)

= *n*_*fu *_+ (*n *- *f*(*Iα*^-1^) + *n*_*fu *_- *n*_*fi*_) + *n*_*fi *_    (by Claim 1)

= *n *- *f*(*Iα*^-1^)

As mentioned before, *χ*(*α*) - 1 and *χ*(*I*) - 1 are the lower bounds of the numbers of fusions and fissions, respectively, required in any optimal series of rearrangement events for transforming *α *into *I*. Hence, the number of 2-cycles in Φ that function as the fusions and fissions is minimum. Along with that Φ = *Iα*^-1 ^can be expressed as a product of *n *- *f*(*Iα*^-1^) 2-cycles, we thus conclude that Φ is an optimal series of the events that transform *α *into *I *with first *n*_*fu *_fusions, then *n*_*bi *_block-interchanges and finally *n*_*fi *_fissions, where *n*_*fu *_= *χ*(*α*) -1, nbi=n−f(I′α′−1)2=n−f(Iα−1)−nfu−nfi2
 MathType@MTEF@5@5@+=feaafiart1ev1aaatCvAUfKttLearuWrP9MDH5MBPbIqV92AaeXatLxBI9gBaebbnrfifHhDYfgasaacH8akY=wiFfYdH8Gipec8Eeeu0xXdbba9frFj0=OqFfea0dXdd9vqai=hGuQ8kuc9pgc9s8qqaq=dirpe0xb9q8qiLsFr0=vr0=vr0dc8meaabaqaciaacaGaaeqabaqabeGadaaakeaacqWGUbGBdaWgaaWcbaGaemOyaiMaemyAaKgabeaakiabg2da9maalaaabaGaemOBa4MaeyOeI0IaemOzayMaeiikaGIafmysaKKbauaaiiGacuWFXoqygaqbamaaCaaaleqabaaccaGae4NeI0ccbaGae0xmaedaaOGaeiykaKcabaGaeGOmaidaaiabg2da9maalaaabaGaemOBa4MaeyOeI0IaemOzayMaeiikaGIaemysaKKae8xSde2aaWbaaSqabeaacqGFsislcqqFXaqmaaGccqGGPaqkcqGHsislcqWGUbGBdaWgaaWcbaGaemOzayMaemyDauhabeaakiabgkHiTiabd6gaUnaaBaaaleaacqWGMbGzcqWGPbqAaeqaaaGcbaGaeGOmaidaaaaa@53DC@, and *n*_*fi *_= *χ*(*I*) - 1.

**Lemma 7 ***There is an optimal series of the events needed to transform α into I in a canonical order such that all n_*fu *_fusions come before all n_*bi *_block-interchanges that come before all n_*fi *_fissions, where n*_*fu *_= *χ*(*α*) - 1, nbi=n−f(Iα−1)−nfu−nfi2
 MathType@MTEF@5@5@+=feaafiart1ev1aaatCvAUfKttLearuWrP9MDH5MBPbIqV92AaeXatLxBI9gBaebbnrfifHhDYfgasaacH8akY=wiFfYdH8Gipec8Eeeu0xXdbba9frFj0=OqFfea0dXdd9vqai=hGuQ8kuc9pgc9s8qqaq=dirpe0xb9q8qiLsFr0=vr0=vr0dc8meaabaqaciaacaGaaeqabaqabeGadaaakeaacqWGUbGBdaWgaaWcbaGaemOyaiMaemyAaKgabeaakiabg2da9maalaaabaGaemOBa4MaeyOeI0IaemOzayMaeiikaGIaemysaKecciGae8xSde2aaWbaaSqabeaaiiaacqGFsisltCvAUfeBSjuyZL2yd9gzLbvyNv2CaeHbwvMCKfMBHbaceaGaa0xmaaaakiabcMcaPiabgkHiTiabd6gaUnaaBaaaleaacqWGMbGzcqWG1bqDaeqaaOGaeyOeI0IaemOBa42aaSbaaSqaaiabdAgaMjabdMgaPbqabaaakeaacqaIYaGmaaaaaa@51FA@*and n*_*fi *_= *χ*(*I*) - 1.

Let us take *α *= (1, 2,10) (11, 8, 9, 3, 6) (7, 4, 5, 12) and *I *= (1, 2, 3) (4, 5) (6, 7, 8) (9, 10, 11, 12) for an example. It should be straightforward to see that G
 MathType@MTEF@5@5@+=feaafiart1ev1aaatCvAUfKttLearuWrP9MDH5MBPbIqV92AaeXatLxBI9gBamrtHrhAL1wy0L2yHvtyaeHbnfgDOvwBHrxAJfwnaebbnrfifHhDYfgasaacH8akY=wiFfYdH8Gipec8Eeeu0xXdbba9frFj0=OqFfea0dXdd9vqai=hGuQ8kuc9pgc9s8qqaq=dirpe0xb9q8qiLsFr0=vr0=vr0dc8meaabaqaciaacaGaaeqabaWaaeGaeaaakeaaimaacqWFge=raaa@382D@(*α*,*I*) is a connected bipartite graph with *χ*(*α*) = 3 and *χ*(*I*) = 4, and *Iα*^-1 ^= (1, 11, 7, 9, 6) (3, 10) (4, 8, 12) and hence *f*(*Iα^-2^*) = 5, since two 1-cycles (i.e., (2) and (5)) are not explicitly shown. First, we are to find two 2-cycles *φ*_1 _and *φ*_2 _(since *n*_*fu *_= *χ*(*α*) -1 = 2) from *Iα*^-1 ^to transform genome *α *with three chromosomes into genome *α*' with exactly one chromosome. To this purpose, we let *φ*_1 _= (3,10) and *φ*_2 _= (4,8), since *Iα*^-1 ^= (1, 11, 7, 9, 6) (4, 12) (4, 8) (3, 10). Then by Claim 1, *α*' = *φ*_2_*φ*_1_*α *= (4, 5, 12, 7, 8, 9, 10, 1, 2, 3, 6, 11) and *Iα*'^-1 ^= *Iα*^-1^*φ*_1_*φ*_2 _= (1, 11, 7, 9, 6) (4, 12). Next, we need to find three 2-cycles *ψ*_1_, *ψ*_2 _and *ψ*_3 _(since *n*_*fi *_= *χ*(*I*) - 1 = 3) from *α*'*I*^-1^, which is equal to (*Iα*'^-1^)^-1 ^= (6, 9, 7, 11, 1) (12, 4) = (1, 7, 11) (1, 9) (1, 6) (12, 4), to transform *I *into *I*' with only one chromosome. By letting *ψ*_1 _= (12, 4), *ψ*_2 _= (1, 6) and *ψ*_3 _= (1, 9), we have *I*' = *ψ*_3_*ψ*_2_*ψ*_1 _= (1, 2, 3, 6, 7, 8, 9, 10, 11, 4, 5, 12) and *α*'*I*'^-1 ^= *α*'*I*^-1^*ψ*_1_*ψ*_2_*ψ*_3 _= (1, 7, 11) according to Claim 2. Finally, we will find two 2-cycles τ11
 MathType@MTEF@5@5@+=feaafiart1ev1aaatCvAUfKttLearuWrP9MDH5MBPbIqV92AaeXatLxBI9gBaebbnrfifHhDYfgasaacH8akY=wiFfYdH8Gipec8Eeeu0xXdbba9frFj0=OqFfea0dXdd9vqai=hGuQ8kuc9pgc9s8qqaq=dirpe0xb9q8qiLsFr0=vr0=vr0dc8meaabaqaciaacaGaaeqabaqabeGadaaakeaaiiGacqWFepaDdaqhaaWcbaGaeeymaedabaGaeeymaedaaaaa@3077@ and τ12
 MathType@MTEF@5@5@+=feaafiart1ev1aaatCvAUfKttLearuWrP9MDH5MBPbIqV92AaeXatLxBI9gBaebbnrfifHhDYfgasaacH8akY=wiFfYdH8Gipec8Eeeu0xXdbba9frFj0=OqFfea0dXdd9vqai=hGuQ8kuc9pgc9s8qqaq=dirpe0xb9q8qiLsFr0=vr0=vr0dc8meaabaqaciaacaGaaeqabaqabeGadaaakeaaiiGacqWFepaDdaqhaaWcbaGaeeymaedabaGaeeOmaidaaaaa@3079@ (since *n *- *f*(*Iα*^-1^) - *n*_*fu *_- *n*_*fi *_= 12 - 5 - 2 - 3 = 2) from *I*'*α*'^-1 ^that act as a block-interchange to transform *α*' into *I*', where *I*'*α*'^-1 ^= (*α*'*I*'^-1^)^-1 ^= (11, 7, 1) = (11, 1) (11, 7). By letting τ11
 MathType@MTEF@5@5@+=feaafiart1ev1aaatCvAUfKttLearuWrP9MDH5MBPbIqV92AaeXatLxBI9gBaebbnrfifHhDYfgasaacH8akY=wiFfYdH8Gipec8Eeeu0xXdbba9frFj0=OqFfea0dXdd9vqai=hGuQ8kuc9pgc9s8qqaq=dirpe0xb9q8qiLsFr0=vr0=vr0dc8meaabaqaciaacaGaaeqabaqabeGadaaakeaaiiGacqWFepaDdaqhaaWcbaGaeeymaedabaGaeeymaedaaaaa@3077@ = (11, 7) and τ12
 MathType@MTEF@5@5@+=feaafiart1ev1aaatCvAUfKttLearuWrP9MDH5MBPbIqV92AaeXatLxBI9gBaebbnrfifHhDYfgasaacH8akY=wiFfYdH8Gipec8Eeeu0xXdbba9frFj0=OqFfea0dXdd9vqai=hGuQ8kuc9pgc9s8qqaq=dirpe0xb9q8qiLsFr0=vr0=vr0dc8meaabaqaciaacaGaaeqabaqabeGadaaakeaaiiGacqWFepaDdaqhaaWcbaGaeeymaedabaGaeeOmaidaaaaa@3079@ = (11, 1), we have τ12
 MathType@MTEF@5@5@+=feaafiart1ev1aaatCvAUfKttLearuWrP9MDH5MBPbIqV92AaeXatLxBI9gBaebbnrfifHhDYfgasaacH8akY=wiFfYdH8Gipec8Eeeu0xXdbba9frFj0=OqFfea0dXdd9vqai=hGuQ8kuc9pgc9s8qqaq=dirpe0xb9q8qiLsFr0=vr0=vr0dc8meaabaqaciaacaGaaeqabaqabeGadaaakeaaiiGacqWFepaDdaqhaaWcbaGaeeymaedabaGaeeOmaidaaaaa@3079@τ11
 MathType@MTEF@5@5@+=feaafiart1ev1aaatCvAUfKttLearuWrP9MDH5MBPbIqV92AaeXatLxBI9gBaebbnrfifHhDYfgasaacH8akY=wiFfYdH8Gipec8Eeeu0xXdbba9frFj0=OqFfea0dXdd9vqai=hGuQ8kuc9pgc9s8qqaq=dirpe0xb9q8qiLsFr0=vr0=vr0dc8meaabaqaciaacaGaaeqabaqabeGadaaakeaaiiGacqWFepaDdaqhaaWcbaGaeeymaedabaGaeeymaedaaaaa@3077@*α*' = (11, 1) (11, 7) (4, 5, 12, 7, 8, 9, 10, 1, 2, 3, 6, 11) = (11, 4, 5, 12, 1, 2, 3, 6, 7, 8, 9, 10), which indeed equals *I*'. Consequently, we find an optimal series of events Φ= *ψ*_1_*ψ*_2_*ψ*_3_τ12
 MathType@MTEF@5@5@+=feaafiart1ev1aaatCvAUfKttLearuWrP9MDH5MBPbIqV92AaeXatLxBI9gBaebbnrfifHhDYfgasaacH8akY=wiFfYdH8Gipec8Eeeu0xXdbba9frFj0=OqFfea0dXdd9vqai=hGuQ8kuc9pgc9s8qqaq=dirpe0xb9q8qiLsFr0=vr0=vr0dc8meaabaqaciaacaGaaeqabaqabeGadaaakeaaiiGacqWFepaDdaqhaaWcbaGaeeymaedabaGaeeOmaidaaaaa@3079@τ11
 MathType@MTEF@5@5@+=feaafiart1ev1aaatCvAUfKttLearuWrP9MDH5MBPbIqV92AaeXatLxBI9gBaebbnrfifHhDYfgasaacH8akY=wiFfYdH8Gipec8Eeeu0xXdbba9frFj0=OqFfea0dXdd9vqai=hGuQ8kuc9pgc9s8qqaq=dirpe0xb9q8qiLsFr0=vr0=vr0dc8meaabaqaciaacaGaaeqabaqabeGadaaakeaaiiGacqWFepaDdaqhaaWcbaGaeeymaedabaGaeeymaedaaaaa@3077@*φ*_2_*φ*_1 _that transform *α *into *I *(i.e., Φ*α *= *I*).

Based on the idea above, we have designed Algorithm Sorting-by-ffbi (meaning sorting by fusions, fissions and block-interchanges) to compute the genome rearrangement distance *d*(*α*, *I*) between two given circular multi-chromosomal genomes *α *and *I*, and also to generate an optimal scenario of the required rearrangement events in a canonical order. In Algorithm Sorting-by-ffbi, the purpose of Step 2.3.3 (respectively, Step 2.4.4) is to find two numbers *x *and *y *that are both in some cycle of *γ *= *J*_*i*_βi−1
 MathType@MTEF@5@5@+=feaafiart1ev1aaatCvAUfKttLearuWrP9MDH5MBPbIqV92AaeXatLxBI9gBaebbnrfifHhDYfgasaacH8akY=wiFfYdH8Gipec8Eeeu0xXdbba9frFj0=OqFfea0dXdd9vqai=hGuQ8kuc9pgc9s8qqaq=dirpe0xb9q8qiLsFr0=vr0=vr0dc8meaabaqaciaacaGaaeqabaqabeGadaaakeaaiiGacqWFYoGydaqhaaWcbaGaemyAaKgabaGaeyOeI0IaeGymaedaaaaa@31B9@ (respectively, *γ *= β′iJi−1
 MathType@MTEF@5@5@+=feaafiart1ev1aaatCvAUfKttLearuWrP9MDH5MBPbIqV92AaeXatLxBI9gBaebbnrfifHhDYfgasaacH8akY=wiFfYdH8Gipec8Eeeu0xXdbba9frFj0=OqFfea0dXdd9vqai=hGuQ8kuc9pgc9s8qqaq=dirpe0xb9q8qiLsFr0=vr0=vr0dc8meaabaqaciaacaGaaeqabaqabeGadaaakeaaiiGacuWFYoGygaqbamaaBaaaleaaieGacqGFPbqAaeqaaOGaemOsaO0aa0baaSqaaiabdMgaPbqaaiabgkHiTiabigdaXaaaaaa@3479@), but in different cycles in *β*_*i*_. By Lemma 5, such *x *and *y *exist. In fact, they can be found using the following simple approach. For simplicity, let *γ*_*k *_= (ak1,ak2,…,aklk
 MathType@MTEF@5@5@+=feaafiart1ev1aaatCvAUfKttLearuWrP9MDH5MBPbIqV92AaeXatLxBI9gBaebbnrfifHhDYfgasaacH8akY=wiFfYdH8Gipec8Eeeu0xXdbba9frFj0=OqFfea0dXdd9vqai=hGuQ8kuc9pgc9s8qqaq=dirpe0xb9q8qiLsFr0=vr0=vr0dc8meaabaqaciaacaGaaeqabaqabeGadaaakeaacqWGHbqydaqhaaWcbaGaem4AaSgabaGaeGymaedaaOGaeiilaWIaemyyae2aa0baaSqaaiabdUgaRbqaaiabikdaYaaakiabcYcaSiablAciljabcYcaSiabdggaHnaaDaaaleaacqWGRbWAaeaacqWGSbaBdaWgaaadbaGaem4AaSgabeaaaaaaaa@3DD6@) be a cycle in *γ *that contains two numbers *x *and *y *such that they are in different cycles in *β*_*i*_. Then we only need to check whether ak1
 MathType@MTEF@5@5@+=feaafiart1ev1aaatCvAUfKttLearuWrP9MDH5MBPbIqV92AaeXatLxBI9gBaebbnrfifHhDYfgasaacH8akY=wiFfYdH8Gipec8Eeeu0xXdbba9frFj0=OqFfea0dXdd9vqai=hGuQ8kuc9pgc9s8qqaq=dirpe0xb9q8qiLsFr0=vr0=vr0dc8meaabaqaciaacaGaaeqabaqabeGadaaakeaacqWGHbqydaqhaaWcbaGaem4AaSgabaGaeGymaedaaaaa@3073@ and akj
 MathType@MTEF@5@5@+=feaafiart1ev1aaatCvAUfKttLearuWrP9MDH5MBPbIqV92AaeXatLxBI9gBaebbnrfifHhDYfgasaacH8akY=wiFfYdH8Gipec8Eeeu0xXdbba9frFj0=OqFfea0dXdd9vqai=hGuQ8kuc9pgc9s8qqaq=dirpe0xb9q8qiLsFr0=vr0=vr0dc8meaabaqaciaacaGaaeqabaqabeGadaaakeaacqWGHbqydaqhaaWcbaGaem4AaSgabaGaemOAaOgaaaaa@30E0@, where 2 ≤ *j *≤ *l*_*k*_, are in different cycles in *β*_*i *_or not. If so, we let *x *= ak1
 MathType@MTEF@5@5@+=feaafiart1ev1aaatCvAUfKttLearuWrP9MDH5MBPbIqV92AaeXatLxBI9gBaebbnrfifHhDYfgasaacH8akY=wiFfYdH8Gipec8Eeeu0xXdbba9frFj0=OqFfea0dXdd9vqai=hGuQ8kuc9pgc9s8qqaq=dirpe0xb9q8qiLsFr0=vr0=vr0dc8meaabaqaciaacaGaaeqabaqabeGadaaakeaacqWGHbqydaqhaaWcbaGaem4AaSgabaGaeGymaedaaaaa@3073@ and *y *= akj
 MathType@MTEF@5@5@+=feaafiart1ev1aaatCvAUfKttLearuWrP9MDH5MBPbIqV92AaeXatLxBI9gBaebbnrfifHhDYfgasaacH8akY=wiFfYdH8Gipec8Eeeu0xXdbba9frFj0=OqFfea0dXdd9vqai=hGuQ8kuc9pgc9s8qqaq=dirpe0xb9q8qiLsFr0=vr0=vr0dc8meaabaqaciaacaGaaeqabaqabeGadaaakeaacqWGHbqydaqhaaWcbaGaem4AaSgabaGaemOAaOgaaaaa@30E0@. The reason is as follows. Suppose that both ak1
 MathType@MTEF@5@5@+=feaafiart1ev1aaatCvAUfKttLearuWrP9MDH5MBPbIqV92AaeXatLxBI9gBaebbnrfifHhDYfgasaacH8akY=wiFfYdH8Gipec8Eeeu0xXdbba9frFj0=OqFfea0dXdd9vqai=hGuQ8kuc9pgc9s8qqaq=dirpe0xb9q8qiLsFr0=vr0=vr0dc8meaabaqaciaacaGaaeqabaqabeGadaaakeaacqWGHbqydaqhaaWcbaGaem4AaSgabaGaeGymaedaaaaa@3073@ and akj
 MathType@MTEF@5@5@+=feaafiart1ev1aaatCvAUfKttLearuWrP9MDH5MBPbIqV92AaeXatLxBI9gBaebbnrfifHhDYfgasaacH8akY=wiFfYdH8Gipec8Eeeu0xXdbba9frFj0=OqFfea0dXdd9vqai=hGuQ8kuc9pgc9s8qqaq=dirpe0xb9q8qiLsFr0=vr0=vr0dc8meaabaqaciaacaGaaeqabaqabeGadaaakeaacqWGHbqydaqhaaWcbaGaem4AaSgabaGaemOAaOgaaaaa@30E0@ for all 2 ≤ *j *≤ *l*_*k *_are in the same cycle in *β*_*i*_. Then all of numbers ak1,ak2,…,aklk
 MathType@MTEF@5@5@+=feaafiart1ev1aaatCvAUfKttLearuWrP9MDH5MBPbIqV92AaeXatLxBI9gBaebbnrfifHhDYfgasaacH8akY=wiFfYdH8Gipec8Eeeu0xXdbba9frFj0=OqFfea0dXdd9vqai=hGuQ8kuc9pgc9s8qqaq=dirpe0xb9q8qiLsFr0=vr0=vr0dc8meaabaqaciaacaGaaeqabaqabeGadaaakeaacqWGHbqydaqhaaWcbaGaem4AaSgabaGaeGymaedaaOGaeiilaWIaemyyae2aa0baaSqaaiabdUgaRbqaaiabikdaYaaakiabcYcaSiablAciljabcYcaSiabdggaHnaaDaaaleaacqWGRbWAaeaacqWGSbaBdaWgaaadbaGaem4AaSgabeaaaaaaaa@3DD6@ in *γ*_*k *_are in the same cycle in *β*_*i*_, which contradicts the above assumption that *γ*_*k *_contains *x *and *y *that are in different cycles in *β*_*i*_.

#### Algorithm sorting-by-ffbi

**Input: **Two circular multi-chromosomal genomes *α *and *I*.

**Output: ***d*(*α*,*I*) and a minimum series Φ of events required to transform *α *into *I*.

**1:**   Find all connected components C
 MathType@MTEF@5@5@+=feaafiart1ev1aaatCvAUfKttLearuWrP9MDH5MBPbIqV92AaeXatLxBI9gBamrtHrhAL1wy0L2yHvtyaeHbnfgDOvwBHrxAJfwnaebbnrfifHhDYfgasaacH8akY=wiFfYdH8Gipec8Eeeu0xXdbba9frFj0=OqFfea0dXdd9vqai=hGuQ8kuc9pgc9s8qqaq=dirpe0xb9q8qiLsFr0=vr0=vr0dc8meaabaqaciaacaGaaeqabaWaaeGaeaaakeaaimaacqWFce=qaaa@3825@_*1*_, C
 MathType@MTEF@5@5@+=feaafiart1ev1aaatCvAUfKttLearuWrP9MDH5MBPbIqV92AaeXatLxBI9gBamrtHrhAL1wy0L2yHvtyaeHbnfgDOvwBHrxAJfwnaebbnrfifHhDYfgasaacH8akY=wiFfYdH8Gipec8Eeeu0xXdbba9frFj0=OqFfea0dXdd9vqai=hGuQ8kuc9pgc9s8qqaq=dirpe0xb9q8qiLsFr0=vr0=vr0dc8meaabaqaciaacaGaaeqabaWaaeGaeaaakeaaimaacqWFce=qaaa@3825@_*2*_,...,C
 MathType@MTEF@5@5@+=feaafiart1ev1aaatCvAUfKttLearuWrP9MDH5MBPbIqV92AaeXatLxBI9gBamrtHrhAL1wy0L2yHvtyaeHbnfgDOvwBHrxAJfwnaebbnrfifHhDYfgasaacH8akY=wiFfYdH8Gipec8Eeeu0xXdbba9frFj0=OqFfea0dXdd9vqai=hGuQ8kuc9pgc9s8qqaq=dirpe0xb9q8qiLsFr0=vr0=vr0dc8meaabaqaciaacaGaaeqabaWaaeGaeaaakeaaimaacqWFce=qaaa@3825@_*ω *_in graph G
 MathType@MTEF@5@5@+=feaafiart1ev1aaatCvAUfKttLearuWrP9MDH5MBPbIqV92AaeXatLxBI9gBamrtHrhAL1wy0L2yHvtyaeHbnfgDOvwBHrxAJfwnaebbnrfifHhDYfgasaacH8akY=wiFfYdH8Gipec8Eeeu0xXdbba9frFj0=OqFfea0dXdd9vqai=hGuQ8kuc9pgc9s8qqaq=dirpe0xb9q8qiLsFr0=vr0=vr0dc8meaabaqaciaacaGaaeqabaWaaeGaeaaakeaaimaacqWFge=raaa@382D@(*α*,*I*);

      /* Denote by *n*_*i*_ the number of genes in C
 MathType@MTEF@5@5@+=feaafiart1ev1aaatCvAUfKttLearuWrP9MDH5MBPbIqV92AaeXatLxBI9gBamrtHrhAL1wy0L2yHvtyaeHbnfgDOvwBHrxAJfwnaebbnrfifHhDYfgasaacH8akY=wiFfYdH8Gipec8Eeeu0xXdbba9frFj0=OqFfea0dXdd9vqai=hGuQ8kuc9pgc9s8qqaq=dirpe0xb9q8qiLsFr0=vr0=vr0dc8meaabaqaciaacaGaaeqabaWaaeGaeaaakeaaimaacqWFce=qaaa@3825@_*i*_. */

**2:**   **for **each C
 MathType@MTEF@5@5@+=feaafiart1ev1aaatCvAUfKttLearuWrP9MDH5MBPbIqV92AaeXatLxBI9gBamrtHrhAL1wy0L2yHvtyaeHbnfgDOvwBHrxAJfwnaebbnrfifHhDYfgasaacH8akY=wiFfYdH8Gipec8Eeeu0xXdbba9frFj0=OqFfea0dXdd9vqai=hGuQ8kuc9pgc9s8qqaq=dirpe0xb9q8qiLsFr0=vr0=vr0dc8meaabaqaciaacaGaaeqabaWaaeGaeaaakeaaimaacqWFce=qaaa@3825@_*i*_, 1 ≤ *i *≤ *ω*,**do**

         /* Denote by *β*_*i *_(resp. *J*_*i*_) the collection of chromosomes in *α *(resp. *I*) whose corresponding vertices are in C
 MathType@MTEF@5@5@+=feaafiart1ev1aaatCvAUfKttLearuWrP9MDH5MBPbIqV92AaeXatLxBI9gBamrtHrhAL1wy0L2yHvtyaeHbnfgDOvwBHrxAJfwnaebbnrfifHhDYfgasaacH8akY=wiFfYdH8Gipec8Eeeu0xXdbba9frFj0=OqFfea0dXdd9vqai=hGuQ8kuc9pgc9s8qqaq=dirpe0xb9q8qiLsFr0=vr0=vr0dc8meaabaqaciaacaGaaeqabaWaaeGaeaaakeaaimaacqWFce=qaaa@3825@_*i*_. */

**2.1:**   Compute *J*_*i*_βi−1
 MathType@MTEF@5@5@+=feaafiart1ev1aaatCvAUfKttLearuWrP9MDH5MBPbIqV92AaeXatLxBI9gBaebbnrfifHhDYfgasaacH8akY=wiFfYdH8Gipec8Eeeu0xXdbba9frFj0=OqFfea0dXdd9vqai=hGuQ8kuc9pgc9s8qqaq=dirpe0xb9q8qiLsFr0=vr0=vr0dc8meaabaqaciaacaGaaeqabaqabeGadaaakeaaiiGacqWFYoGydaqhaaWcbaGaemyAaKgabaGaeyOeI0IaeGymaedaaaaa@31B9@ and let *γ *= *J*_*i*_βi−1
 MathType@MTEF@5@5@+=feaafiart1ev1aaatCvAUfKttLearuWrP9MDH5MBPbIqV92AaeXatLxBI9gBaebbnrfifHhDYfgasaacH8akY=wiFfYdH8Gipec8Eeeu0xXdbba9frFj0=OqFfea0dXdd9vqai=hGuQ8kuc9pgc9s8qqaq=dirpe0xb9q8qiLsFr0=vr0=vr0dc8meaabaqaciaacaGaaeqabaqabeGadaaakeaaiiGacqWFYoGydaqhaaWcbaGaemyAaKgabaGaeyOeI0IaeGymaedaaaaa@31B9@;

**2.2:**   *n*_*fu *_= *χ*(*β*_*i*_) - 1, *n*_*fi *_= *χ*(*J*_*i*_) -1, nbi=ni−f(γ)−nfu−nfi2
 MathType@MTEF@5@5@+=feaafiart1ev1aaatCvAUfKttLearuWrP9MDH5MBPbIqV92AaeXatLxBI9gBaebbnrfifHhDYfgasaacH8akY=wiFfYdH8Gipec8Eeeu0xXdbba9frFj0=OqFfea0dXdd9vqai=hGuQ8kuc9pgc9s8qqaq=dirpe0xb9q8qiLsFr0=vr0=vr0dc8meaabaqaciaacaGaaeqabaqabeGadaaakeaacqWGUbGBdaWgaaWcbaGaemOyaiMaemyAaKgabeaakiabg2da9maalaaabaGaemOBa42aaSbaaSqaaiabdMgaPbqabaGccqGHsislcqWGMbGzcqGGOaakiiGacqWFZoWzcqGGPaqkcqGHsislcqWGUbGBdaWgaaWcbaGaemOzayMaemyDauhabeaakiabgkHiTiabd6gaUnaaBaaaleaacqWGMbGzcqWGPbqAaeqaaaGcbaGaeGOmaidaaaaa@4617@ and *δ*_*i *_= *n*_*fu *_+ *n*_*bi *_+ *n*_*fi*_;

**2.3:**   **if ***χ*(*β*_*i*_) > 1 **then**   /* To compute *φ*_1_, *φ*_2_,...,φnfu
 MathType@MTEF@5@5@+=feaafiart1ev1aaatCvAUfKttLearuWrP9MDH5MBPbIqV92AaeXatLxBI9gBaebbnrfifHhDYfgasaacH8akY=wiFfYdH8Gipec8Eeeu0xXdbba9frFj0=OqFfea0dXdd9vqai=hGuQ8kuc9pgc9s8qqaq=dirpe0xb9q8qiLsFr0=vr0=vr0dc8meaabaqaciaacaGaaeqabaqabeGadaaakeaaiiGacqWFgpGzdaWgaaWcbaGaemOBa42aaSbaaWqaaiabdAgaMjabdwha1bqabaaaleqaaaaa@32FD@ */

**2.3.1:**      **for **each cycle of *β*_*i *_**do**

         Create a set to contain all the numbers in this cycle;

   **endfor**

**2.3.2:**   /* Let *γ*_1_*γ*_2 _... *γ*_*p *_be the cycle decomposition of the current *γ *and

   let *γ*_*q *_= (aq1,aq2,…,aqlq
 MathType@MTEF@5@5@+=feaafiart1ev1aaatCvAUfKttLearuWrP9MDH5MBPbIqV92AaeXatLxBI9gBaebbnrfifHhDYfgasaacH8akY=wiFfYdH8Gipec8Eeeu0xXdbba9frFj0=OqFfea0dXdd9vqai=hGuQ8kuc9pgc9s8qqaq=dirpe0xb9q8qiLsFr0=vr0=vr0dc8meaabaqaciaacaGaaeqabaqabeGadaaakeaacWaJaoyyae2aiWiGDaaaleacmcOamWiGdghaXbqaiWiGcWaJaIymaedaaOGaeiilaWIaemyyae2aa0baaSqaaiabdghaXbqaaiabikdaYaaakiabcYcaSiablAciljabcYcaSiabdggaHnaaDaaaleaacqWGXbqCaeaacqWGSbaBdaWgaaadbaGaemyCaehabeaaaaaaaa@447E@), where 1 ≤ *q *≤ *p *and *l*_*q *_≥ 2 */

   *k *= 1 and *h *= 2;

**2.3.3:**   **for ***j *= 1 to *n*_*fu *_**do**

      *S *= find-set (ak1
 MathType@MTEF@5@5@+=feaafiart1ev1aaatCvAUfKttLearuWrP9MDH5MBPbIqV92AaeXatLxBI9gBaebbnrfifHhDYfgasaacH8akY=wiFfYdH8Gipec8Eeeu0xXdbba9frFj0=OqFfea0dXdd9vqai=hGuQ8kuc9pgc9s8qqaq=dirpe0xb9q8qiLsFr0=vr0=vr0dc8meaabaqaciaacaGaaeqabaqabeGadaaakeaacqWGHbqydaqhaaWcbaGaem4AaSgabaGaeGymaedaaaaa@3073@);

      **while ***S *= find-set(akh
 MathType@MTEF@5@5@+=feaafiart1ev1aaatCvAUfKttLearuWrP9MDH5MBPbIqV92AaeXatLxBI9gBaebbnrfifHhDYfgasaacH8akY=wiFfYdH8Gipec8Eeeu0xXdbba9frFj0=OqFfea0dXdd9vqai=hGuQ8kuc9pgc9s8qqaq=dirpe0xb9q8qiLsFr0=vr0=vr0dc8meaabaqaciaacaGaaeqabaqabeGadaaakeaacqWGHbqydaqhaaWcbaGaem4AaSgabaGaemiAaGgaaaaa@30DC@)) **do**

         **if ***h *<*l*_*k *_**then ***h *= *h *+ 1; **else ***k *= *k *+ 1, *h *= 2 and *S *= findset (ak1
 MathType@MTEF@5@5@+=feaafiart1ev1aaatCvAUfKttLearuWrP9MDH5MBPbIqV92AaeXatLxBI9gBaebbnrfifHhDYfgasaacH8akY=wiFfYdH8Gipec8Eeeu0xXdbba9frFj0=OqFfea0dXdd9vqai=hGuQ8kuc9pgc9s8qqaq=dirpe0xb9q8qiLsFr0=vr0=vr0dc8meaabaqaciaacaGaaeqabaqabeGadaaakeaacqWGHbqydaqhaaWcbaGaem4AaSgabaGaeGymaedaaaaa@3073@);

      **endwhile**

      *x *= ak1
 MathType@MTEF@5@5@+=feaafiart1ev1aaatCvAUfKttLearuWrP9MDH5MBPbIqV92AaeXatLxBI9gBaebbnrfifHhDYfgasaacH8akY=wiFfYdH8Gipec8Eeeu0xXdbba9frFj0=OqFfea0dXdd9vqai=hGuQ8kuc9pgc9s8qqaq=dirpe0xb9q8qiLsFr0=vr0=vr0dc8meaabaqaciaacaGaaeqabaqabeGadaaakeaacqWGHbqydaqhaaWcbaGaem4AaSgabaGaeGymaedaaaaa@3073@ and *y *= akh
 MathType@MTEF@5@5@+=feaafiart1ev1aaatCvAUfKttLearuWrP9MDH5MBPbIqV92AaeXatLxBI9gBaebbnrfifHhDYfgasaacH8akY=wiFfYdH8Gipec8Eeeu0xXdbba9frFj0=OqFfea0dXdd9vqai=hGuQ8kuc9pgc9s8qqaq=dirpe0xb9q8qiLsFr0=vr0=vr0dc8meaabaqaciaacaGaaeqabaqabeGadaaakeaacqWGHbqydaqhaaWcbaGaem4AaSgabaGaemiAaGgaaaaa@30DC@;

      *φ*_*j *_= (*x*, *y*) and union(*x*, *y*);

   **endfor**

**2.3.4:**   β′i
 MathType@MTEF@5@5@+=feaafiart1ev1aaatCvAUfKttLearuWrP9MDH5MBPbIqV92AaeXatLxBI9gBaebbnrfifHhDYfgasaacH8akY=wiFfYdH8Gipec8Eeeu0xXdbba9frFj0=OqFfea0dXdd9vqai=hGuQ8kuc9pgc9s8qqaq=dirpe0xb9q8qiLsFr0=vr0=vr0dc8meaabaqaciaacaGaaeqabaqabeGadaaakeaaiiGacuWFYoGygaqbamaaBaaaleaaieGacqGFPbqAaeqaaaaa@2FED@ = φnfu
 MathType@MTEF@5@5@+=feaafiart1ev1aaatCvAUfKttLearuWrP9MDH5MBPbIqV92AaeXatLxBI9gBaebbnrfifHhDYfgasaacH8akY=wiFfYdH8Gipec8Eeeu0xXdbba9frFj0=OqFfea0dXdd9vqai=hGuQ8kuc9pgc9s8qqaq=dirpe0xb9q8qiLsFr0=vr0=vr0dc8meaabaqaciaacaGaaeqabaqabeGadaaakeaaiiGacqWFgpGzdaWgaaWcbaGaemOBa42aaSbaaWqaaiabdAgaMjabdwha1bqabaaaleqaaaaa@32FD@φnfu−1
 MathType@MTEF@5@5@+=feaafiart1ev1aaatCvAUfKttLearuWrP9MDH5MBPbIqV92AaeXatLxBI9gBaebbnrfifHhDYfgasaacH8akY=wiFfYdH8Gipec8Eeeu0xXdbba9frFj0=OqFfea0dXdd9vqai=hGuQ8kuc9pgc9s8qqaq=dirpe0xb9q8qiLsFr0=vr0=vr0dc8meaabaqaciaacaGaaeqabaqabeGadaaakeaaiiGacqWFgpGzdaWgaaWcbaGaemOBa42aaSbaaWqaaiabdAgaMjabdwha1bqabaWccqGHsislcqaIXaqmaeqaaaaa@34DA@...*φ*_1_*β*_*i *_and *γ *= *γφ*_1_*φ*_2_...φnfu
 MathType@MTEF@5@5@+=feaafiart1ev1aaatCvAUfKttLearuWrP9MDH5MBPbIqV92AaeXatLxBI9gBaebbnrfifHhDYfgasaacH8akY=wiFfYdH8Gipec8Eeeu0xXdbba9frFj0=OqFfea0dXdd9vqai=hGuQ8kuc9pgc9s8qqaq=dirpe0xb9q8qiLsFr0=vr0=vr0dc8meaabaqaciaacaGaaeqabaqabeGadaaakeaaiiGacqWFgpGzdaWgaaWcbaGaemOBa42aaSbaaWqaaiabdAgaMjabdwha1bqabaaaleqaaaaa@32FD@;   /* Currently, *γ *is *J*_i_β′i−1
 MathType@MTEF@5@5@+=feaafiart1ev1aaatCvAUfKttLearuWrP9MDH5MBPbIqV92AaeXatLxBI9gBaebbnrfifHhDYfgasaacH8akY=wiFfYdH8Gipec8Eeeu0xXdbba9frFj0=OqFfea0dXdd9vqai=hGuQ8kuc9pgc9s8qqaq=dirpe0xb9q8qiLsFr0=vr0=vr0dc8meaabaqaciaacaGaaeqabaqabeGadaaakeaaiiGacuWFYoGygaqbamaaDaaaleaacqWGPbqAaeaacqGHsislcqaIXaqmaaaaaa@31C5@ */

   **endif**

**2.4:**   **if ***χ*(*J*_*i*_) > 1 **then**/* To compute *ψ*_1_, *ψ*_2_,...,ψnfi
 MathType@MTEF@5@5@+=feaafiart1ev1aaatCvAUfKttLearuWrP9MDH5MBPbIqV92AaeXatLxBI9gBaebbnrfifHhDYfgasaacH8akY=wiFfYdH8Gipec8Eeeu0xXdbba9frFj0=OqFfea0dXdd9vqai=hGuQ8kuc9pgc9s8qqaq=dirpe0xb9q8qiLsFr0=vr0=vr0dc8meaabaqaciaacaGaaeqabaqabeGadaaakeaaiiGacqWFipqEdaWgaaWcbaGaemOBa42aaSbaaWqaaiabdAgaMjabdMgaPbqabaaaleqaaaaa@32FA@ */

**2.4.1:**      *γ *= *γ*^-1^/* New *γ *becomes β′iJi−1
 MathType@MTEF@5@5@+=feaafiart1ev1aaatCvAUfKttLearuWrP9MDH5MBPbIqV92AaeXatLxBI9gBaebbnrfifHhDYfgasaacH8akY=wiFfYdH8Gipec8Eeeu0xXdbba9frFj0=OqFfea0dXdd9vqai=hGuQ8kuc9pgc9s8qqaq=dirpe0xb9q8qiLsFr0=vr0=vr0dc8meaabaqaciaacaGaaeqabaqabeGadaaakeaaiiGacuWFYoGygaqbamaaBaaaleaaieGacqGFPbqAaeqaaOGaemOsaO0aa0baaSqaaiabdMgaPbqaaiabgkHiTiabigdaXaaaaaa@3479@ */

**2.4.2:**   **for **each cycle of *J*_*i*_** do**

         Create a set to contain all the numbers in this cycle;

      **endfor**

**2.4.3:**   /* Let *γ*_1_*γ*_2_...*γ*_*p *_be the cycle decomposition of the current *γ *and

      let *γ*_*q *_= (aq1,aq2,…,aqlq
 MathType@MTEF@5@5@+=feaafiart1ev1aaatCvAUfKttLearuWrP9MDH5MBPbIqV92AaeXatLxBI9gBaebbnrfifHhDYfgasaacH8akY=wiFfYdH8Gipec8Eeeu0xXdbba9frFj0=OqFfea0dXdd9vqai=hGuQ8kuc9pgc9s8qqaq=dirpe0xb9q8qiLsFr0=vr0=vr0dc8meaabaqaciaacaGaaeqabaqabeGadaaakeaacWaJaoyyae2aiWiGDaaaleacmcOamWiGdghaXbqaiWiGcWaJaIymaedaaOGaeiilaWIaemyyae2aa0baaSqaaiabdghaXbqaaiabikdaYaaakiabcYcaSiablAciljabcYcaSiabdggaHnaaDaaaleaacqWGXbqCaeaacqWGSbaBdaWgaaadbaGaemyCaehabeaaaaaaaa@447E@), where 1 ≤ *q *≤ *p *and *l*_*q *_≥ 2 */

      *k *= 1 and *h *= 2;

**2.4.4:**   **for ***j *= 1 to *n*_*fi *_**do**

      *S *= find-set(ak1
 MathType@MTEF@5@5@+=feaafiart1ev1aaatCvAUfKttLearuWrP9MDH5MBPbIqV92AaeXatLxBI9gBaebbnrfifHhDYfgasaacH8akY=wiFfYdH8Gipec8Eeeu0xXdbba9frFj0=OqFfea0dXdd9vqai=hGuQ8kuc9pgc9s8qqaq=dirpe0xb9q8qiLsFr0=vr0=vr0dc8meaabaqaciaacaGaaeqabaqabeGadaaakeaacqWGHbqydaqhaaWcbaGaem4AaSgabaGaeGymaedaaaaa@3073@);

      **while **(*S *= find-set(akh
 MathType@MTEF@5@5@+=feaafiart1ev1aaatCvAUfKttLearuWrP9MDH5MBPbIqV92AaeXatLxBI9gBaebbnrfifHhDYfgasaacH8akY=wiFfYdH8Gipec8Eeeu0xXdbba9frFj0=OqFfea0dXdd9vqai=hGuQ8kuc9pgc9s8qqaq=dirpe0xb9q8qiLsFr0=vr0=vr0dc8meaabaqaciaacaGaaeqabaqabeGadaaakeaacqWGHbqydaqhaaWcbaGaem4AaSgabaGaemiAaGgaaaaa@30DC@)) **do**

         **if ***h *<*I*_*k *_**then ***h *= *h *+ 1; **else ***k *= *k *+ 1,*h *= 2 and *S *= find-set(ak1
 MathType@MTEF@5@5@+=feaafiart1ev1aaatCvAUfKttLearuWrP9MDH5MBPbIqV92AaeXatLxBI9gBaebbnrfifHhDYfgasaacH8akY=wiFfYdH8Gipec8Eeeu0xXdbba9frFj0=OqFfea0dXdd9vqai=hGuQ8kuc9pgc9s8qqaq=dirpe0xb9q8qiLsFr0=vr0=vr0dc8meaabaqaciaacaGaaeqabaqabeGadaaakeaacqWGHbqydaqhaaWcbaGaem4AaSgabaGaeGymaedaaaaa@3073@);

      **endwhile **

      *x *= ak1
 MathType@MTEF@5@5@+=feaafiart1ev1aaatCvAUfKttLearuWrP9MDH5MBPbIqV92AaeXatLxBI9gBaebbnrfifHhDYfgasaacH8akY=wiFfYdH8Gipec8Eeeu0xXdbba9frFj0=OqFfea0dXdd9vqai=hGuQ8kuc9pgc9s8qqaq=dirpe0xb9q8qiLsFr0=vr0=vr0dc8meaabaqaciaacaGaaeqabaqabeGadaaakeaacqWGHbqydaqhaaWcbaGaem4AaSgabaGaeGymaedaaaaa@3073@ and *y *= akh
 MathType@MTEF@5@5@+=feaafiart1ev1aaatCvAUfKttLearuWrP9MDH5MBPbIqV92AaeXatLxBI9gBaebbnrfifHhDYfgasaacH8akY=wiFfYdH8Gipec8Eeeu0xXdbba9frFj0=OqFfea0dXdd9vqai=hGuQ8kuc9pgc9s8qqaq=dirpe0xb9q8qiLsFr0=vr0=vr0dc8meaabaqaciaacaGaaeqabaqabeGadaaakeaacqWGHbqydaqhaaWcbaGaem4AaSgabaGaemiAaGgaaaaa@30DC@;

      *ψ*_*j *_= (*x*, *y*) and union(*x*, *y*);

   **endfor**

**2.4.5:**    = ψnfi
 MathType@MTEF@5@5@+=feaafiart1ev1aaatCvAUfKttLearuWrP9MDH5MBPbIqV92AaeXatLxBI9gBaebbnrfifHhDYfgasaacH8akY=wiFfYdH8Gipec8Eeeu0xXdbba9frFj0=OqFfea0dXdd9vqai=hGuQ8kuc9pgc9s8qqaq=dirpe0xb9q8qiLsFr0=vr0=vr0dc8meaabaqaciaacaGaaeqabaqabeGadaaakeaaiiGacqWFipqEdaWgaaWcbaGaemOBa42aaSbaaWqaaiabdAgaMjabdMgaPbqabaaaleqaaaaa@32FA@ψnfi−1
 MathType@MTEF@5@5@+=feaafiart1ev1aaatCvAUfKttLearuWrP9MDH5MBPbIqV92AaeXatLxBI9gBaebbnrfifHhDYfgasaacH8akY=wiFfYdH8Gipec8Eeeu0xXdbba9frFj0=OqFfea0dXdd9vqai=hGuQ8kuc9pgc9s8qqaq=dirpe0xb9q8qiLsFr0=vr0=vr0dc8meaabaqaciaacaGaaeqabaqabeGadaaakeaaiiGacqWFipqEdaWgaaWcbaGaemOBa42aaSbaaWqaaiabdAgaMjabdMgaPbqabaWccqGHsislcqaIXaqmaeqaaaaa@34D7@... *ψ*_1_*J*_*i *_and *γ *= *γψ*_1_*ψ*_2_...ψnfi
 MathType@MTEF@5@5@+=feaafiart1ev1aaatCvAUfKttLearuWrP9MDH5MBPbIqV92AaeXatLxBI9gBaebbnrfifHhDYfgasaacH8akY=wiFfYdH8Gipec8Eeeu0xXdbba9frFj0=OqFfea0dXdd9vqai=hGuQ8kuc9pgc9s8qqaq=dirpe0xb9q8qiLsFr0=vr0=vr0dc8meaabaqaciaacaGaaeqabaqabeGadaaakeaaiiGacqWFipqEdaWgaaWcbaGaemOBa42aaSbaaWqaaiabdAgaMjabdMgaPbqabaaaleqaaaaa@32FA@;   /* Currently, *γ *is β′iJ′i−1
 MathType@MTEF@5@5@+=feaafiart1ev1aaatCvAUfKttLearuWrP9MDH5MBPbIqV92AaeXatLxBI9gBaebbnrfifHhDYfgasaacH8akY=wiFfYdH8Gipec8Eeeu0xXdbba9frFj0=OqFfea0dXdd9vqai=hGuQ8kuc9pgc9s8qqaq=dirpe0xb9q8qiLsFr0=vr0=vr0dc8meaabaqaciaacaGaaeqabaqabeGadaaakeaaiiGacuWFYoGygaqbamaaBaaaleaaieGacqGFPbqAaeqaaOGaemOsaO0aa0baaSqaaiabdMgaPbqaaiabgkHiTiabigdaXaaaaaa@3479@ */

   **endif**

**2.5:**   /* To compute τ11,τ12,…,τnbi1,τnbi2
 MathType@MTEF@5@5@+=feaafiart1ev1aaatCvAUfKttLearuWrP9MDH5MBPbIqV92AaeXatLxBI9gBaebbnrfifHhDYfgasaacH8akY=wiFfYdH8Gipec8Eeeu0xXdbba9frFj0=OqFfea0dXdd9vqai=hGuQ8kuc9pgc9s8qqaq=dirpe0xb9q8qiLsFr0=vr0=vr0dc8meaabaqaciaacaGaaeqabaqabeGadaaakeaaiiGacqWFepaDdaqhaaWcbaGaeGymaedabaGaeGymaedaaOGaeiilaWIae8hXdq3aa0baaSqaaiabigdaXaqaaiabikdaYaaakiabcYcaSiablAciljabcYcaSiab=r8a0naaDaaaleaacqWGUbGBdaWgaaadbaGaemOyaiMaemyAaKgabeaaaSqaaiabigdaXaaakiabcYcaSiab=r8a0naaDaaaleaacqWGUbGBdaWgaaadbaGaemOyaiMaemyAaKgabeaaaSqaaiabikdaYaaaaaa@475A@ */

**2.5.1:**   *γ *= *γ*^-1^;/* New *γ *becomes J′i
 MathType@MTEF@5@5@+=feaafiart1ev1aaatCvAUfKttLearuWrP9MDH5MBPbIqV92AaeXatLxBI9gBaebbnrfifHhDYfgasaacH8akY=wiFfYdH8Gipec8Eeeu0xXdbba9frFj0=OqFfea0dXdd9vqai=hGuQ8kuc9pgc9s8qqaq=dirpe0xb9q8qiLsFr0=vr0=vr0dc8meaabaqaciaacaGaaeqabaqabeGadaaakeaacuWGkbGsgaqbamaaBaaaleaacqWGPbqAaeqaaaaa@2F5C@β′i−1
 MathType@MTEF@5@5@+=feaafiart1ev1aaatCvAUfKttLearuWrP9MDH5MBPbIqV92AaeXatLxBI9gBaebbnrfifHhDYfgasaacH8akY=wiFfYdH8Gipec8Eeeu0xXdbba9frFj0=OqFfea0dXdd9vqai=hGuQ8kuc9pgc9s8qqaq=dirpe0xb9q8qiLsFr0=vr0=vr0dc8meaabaqaciaacaGaaeqabaqabeGadaaakeaaiiGacuWFYoGygaqbamaaDaaaleaacqWGPbqAaeaacqGHsislcqaIXaqmaaaaaa@31C5@ */

**2.5.2:**   nbi=ni−f(γ)2
 MathType@MTEF@5@5@+=feaafiart1ev1aaatCvAUfKttLearuWrP9MDH5MBPbIqV92AaeXatLxBI9gBaebbnrfifHhDYfgasaacH8akY=wiFfYdH8Gipec8Eeeu0xXdbba9frFj0=OqFfea0dXdd9vqai=hGuQ8kuc9pgc9s8qqaq=dirpe0xb9q8qiLsFr0=vr0=vr0dc8meaabaqaciaacaGaaeqabaqabeGadaaakeaacqWGUbGBdaWgaaWcbaGaemOyaiMaemyAaKgabeaakiabg2da9maalaaabaGaemOBa42aaSbaaSqaaiabdMgaPbqabaGccqGHsislcqWGMbGzcqGGOaakiiGacqWFZoWzcqGGPaqkaeaacqaIYaGmaaaaaa@3B8F@;

**2.5.3:**   **for ***j *= 1 to *n*_*bi *_**do**

      Arbitrarily choose two adjacent elements *x *and *y *in *γ*;

      /* Let β′i
 MathType@MTEF@5@5@+=feaafiart1ev1aaatCvAUfKttLearuWrP9MDH5MBPbIqV92AaeXatLxBI9gBaebbnrfifHhDYfgasaacH8akY=wiFfYdH8Gipec8Eeeu0xXdbba9frFj0=OqFfea0dXdd9vqai=hGuQ8kuc9pgc9s8qqaq=dirpe0xb9q8qiLsFr0=vr0=vr0dc8meaabaqaciaacaGaaeqabaqabeGadaaakeaaiiGacuWFYoGygaqbamaaBaaaleaaieGacqGFPbqAaeqaaaaa@2FED@ = (*a*_1_,*a*_2_...,ani
 MathType@MTEF@5@5@+=feaafiart1ev1aaatCvAUfKttLearuWrP9MDH5MBPbIqV92AaeXatLxBI9gBaebbnrfifHhDYfgasaacH8akY=wiFfYdH8Gipec8Eeeu0xXdbba9frFj0=OqFfea0dXdd9vqai=hGuQ8kuc9pgc9s8qqaq=dirpe0xb9q8qiLsFr0=vr0=vr0dc8meaabaqaciaacaGaaeqabaqabeGadaaakeaacqWGHbqydaWgaaWcbaGaemOBa42aaSbaaWqaaiabdMgaPbqabaaaleqaaaaa@311B@) */

      Circularly shift (*a*_1_, *a*_2_,...,ani
 MathType@MTEF@5@5@+=feaafiart1ev1aaatCvAUfKttLearuWrP9MDH5MBPbIqV92AaeXatLxBI9gBaebbnrfifHhDYfgasaacH8akY=wiFfYdH8Gipec8Eeeu0xXdbba9frFj0=OqFfea0dXdd9vqai=hGuQ8kuc9pgc9s8qqaq=dirpe0xb9q8qiLsFr0=vr0=vr0dc8meaabaqaciaacaGaaeqabaqabeGadaaakeaacqWGHbqydaWgaaWcbaGaemOBa42aaSbaaWqaaiabdMgaPbqabaaaleqaaaaa@311B@) such that *a*_1 _= *x *and assume *y *= *a*_*k*_;

      τj1
 MathType@MTEF@5@5@+=feaafiart1ev1aaatCvAUfKttLearuWrP9MDH5MBPbIqV92AaeXatLxBI9gBaebbnrfifHhDYfgasaacH8akY=wiFfYdH8Gipec8Eeeu0xXdbba9frFj0=OqFfea0dXdd9vqai=hGuQ8kuc9pgc9s8qqaq=dirpe0xb9q8qiLsFr0=vr0=vr0dc8meaabaqaciaacaGaaeqabaqabeGadaaakeaaiiGacqWFepaDdaqhaaWcbaacbiGae4NAaOgabaGaeeymaedaaaaa@30F1@ = (*x*, *y*);

      **for ***h *= 1 to *n*_*i *_**do**

         index (*a*_*h*_) = *h*;

      **end for**

      Find two adjacent elements *u *and *v *in *γ *(*x*, *y*) such that

      index(*u*) ≤ *k *- 1 and index(*v*) ≥ *k*;

      τj2
 MathType@MTEF@5@5@+=feaafiart1ev1aaatCvAUfKttLearuWrP9MDH5MBPbIqV92AaeXatLxBI9gBaebbnrfifHhDYfgasaacH8akY=wiFfYdH8Gipec8Eeeu0xXdbba9frFj0=OqFfea0dXdd9vqai=hGuQ8kuc9pgc9s8qqaq=dirpe0xb9q8qiLsFr0=vr0=vr0dc8meaabaqaciaacaGaaeqabaqabeGadaaakeaaiiGacqWFepaDdaqhaaWcbaacbiGae4NAaOgabaGaeeOmaidaaaaa@30F3@ = (*u, v*);

      β′i
 MathType@MTEF@5@5@+=feaafiart1ev1aaatCvAUfKttLearuWrP9MDH5MBPbIqV92AaeXatLxBI9gBaebbnrfifHhDYfgasaacH8akY=wiFfYdH8Gipec8Eeeu0xXdbba9frFj0=OqFfea0dXdd9vqai=hGuQ8kuc9pgc9s8qqaq=dirpe0xb9q8qiLsFr0=vr0=vr0dc8meaabaqaciaacaGaaeqabaqabeGadaaakeaaiiGacuWFYoGygaqbamaaBaaaleaaieGacqGFPbqAaeqaaaaa@2FED@ = τj2
 MathType@MTEF@5@5@+=feaafiart1ev1aaatCvAUfKttLearuWrP9MDH5MBPbIqV92AaeXatLxBI9gBaebbnrfifHhDYfgasaacH8akY=wiFfYdH8Gipec8Eeeu0xXdbba9frFj0=OqFfea0dXdd9vqai=hGuQ8kuc9pgc9s8qqaq=dirpe0xb9q8qiLsFr0=vr0=vr0dc8meaabaqaciaacaGaaeqabaqabeGadaaakeaaiiGacqWFepaDdaqhaaWcbaacbiGae4NAaOgabaGaeeOmaidaaaaa@30F3@τj1
 MathType@MTEF@5@5@+=feaafiart1ev1aaatCvAUfKttLearuWrP9MDH5MBPbIqV92AaeXatLxBI9gBaebbnrfifHhDYfgasaacH8akY=wiFfYdH8Gipec8Eeeu0xXdbba9frFj0=OqFfea0dXdd9vqai=hGuQ8kuc9pgc9s8qqaq=dirpe0xb9q8qiLsFr0=vr0=vr0dc8meaabaqaciaacaGaaeqabaqabeGadaaakeaaiiGacqWFepaDdaqhaaWcbaacbiGae4NAaOgabaGaeeymaedaaaaa@30F1@β′i
 MathType@MTEF@5@5@+=feaafiart1ev1aaatCvAUfKttLearuWrP9MDH5MBPbIqV92AaeXatLxBI9gBaebbnrfifHhDYfgasaacH8akY=wiFfYdH8Gipec8Eeeu0xXdbba9frFj0=OqFfea0dXdd9vqai=hGuQ8kuc9pgc9s8qqaq=dirpe0xb9q8qiLsFr0=vr0=vr0dc8meaabaqaciaacaGaaeqabaqabeGadaaakeaaiiGacuWFYoGygaqbamaaBaaaleaaieGacqGFPbqAaeqaaaaa@2FED@ and *γ *= *γ*τj1
 MathType@MTEF@5@5@+=feaafiart1ev1aaatCvAUfKttLearuWrP9MDH5MBPbIqV92AaeXatLxBI9gBaebbnrfifHhDYfgasaacH8akY=wiFfYdH8Gipec8Eeeu0xXdbba9frFj0=OqFfea0dXdd9vqai=hGuQ8kuc9pgc9s8qqaq=dirpe0xb9q8qiLsFr0=vr0=vr0dc8meaabaqaciaacaGaaeqabaqabeGadaaakeaaiiGacqWFepaDdaqhaaWcbaacbiGae4NAaOgabaGaeeymaedaaaaa@30F1@τj2
 MathType@MTEF@5@5@+=feaafiart1ev1aaatCvAUfKttLearuWrP9MDH5MBPbIqV92AaeXatLxBI9gBaebbnrfifHhDYfgasaacH8akY=wiFfYdH8Gipec8Eeeu0xXdbba9frFj0=OqFfea0dXdd9vqai=hGuQ8kuc9pgc9s8qqaq=dirpe0xb9q8qiLsFr0=vr0=vr0dc8meaabaqaciaacaGaaeqabaqabeGadaaakeaaiiGacqWFepaDdaqhaaWcbaacbiGae4NAaOgabaGaeeOmaidaaaaa@30F3@;

   **endfor**

**3:**   Let Φi=ψ1…ψnfiτnbi2τnbi1…τ12τ11φnfu…φ1
 MathType@MTEF@5@5@+=feaafiart1ev1aaatCvAUfKttLearuWrP9MDH5MBPbIqV92AaeXatLxBI9gBaebbnrfifHhDYfgasaacH8akY=wiFfYdH8Gipec8Eeeu0xXdbba9frFj0=OqFfea0dXdd9vqai=hGuQ8kuc9pgc9s8qqaq=dirpe0xb9q8qiLsFr0=vr0=vr0dc8meaabaqaciaacaGaaeqabaqabeGadaaakeaacqqHMoGrdaWgaaWcbaGaemyAaKgabeaakiabg2da9GGaciab=H8a5naaBaaaleaacqaIXaqmaeqaaOGaeSOjGSKae8hYdK3aaSbaaSqaaiabd6gaUnaaBaaameaacqWGMbGzcqWGPbqAaeqaaaWcbeaakiab=r8a0naaDaaaleaacqWGUbGBdaWgaaadbaGaemOyaiMaemyAaKgabeaaaSqaaiabikdaYaaakiab=r8a0naaDaaaleaacqWGUbGBdaWgaaadbaGaemOyaiMaemyAaKgabeaaaSqaaiabigdaXaaakiablAciljab=r8a0naaDaaaleaacqaIXaqmaeaacqaIYaGmaaGccqWFepaDdaqhaaWcbaGaeGymaedabaGaeGymaedaaOGae8NXdy2aaSbaaSqaaiabd6gaUnaaBaaameaacqWGMbGzcqWG1bqDaeqaaaWcbeaakiablAciljab=z8aMnaaBaaaleaacqaIXaqmaeqaaaaa@5C93@ for each 1 ≤ *i *≤ *ω*;.

**4:**   Output *d*(*α*, *I*) = ∑i=1ωδi
 MathType@MTEF@5@5@+=feaafiart1ev1aaatCvAUfKttLearuWrP9MDH5MBPbIqV92AaeXatLxBI9gBaebbnrfifHhDYfgasaacH8akY=wiFfYdH8Gipec8Eeeu0xXdbba9frFj0=OqFfea0dXdd9vqai=hGuQ8kuc9pgc9s8qqaq=dirpe0xb9q8qiLsFr0=vr0=vr0dc8meaabaqaciaacaGaaeqabaqabeGadaaakeaadaaeWaqaaGGaciab=r7aKnaaBaaaleaacqWGPbqAaeqaaaqaaiabdMgaPjabg2da9iabigdaXaqaaiab=L8a3bqdcqGHris5aaaa@36EF@ and Φ = Φ_1_Φ_2_...Φ_*ω*_;

**Theorem 1 ***Given two circular multi-chromosomal genomes α and I over the same gene set E *= {1,2,..., *n*}, *the problem of computing the genome rearrangement distance between α and I using fusions, fissions and block-interchanges can be solved and an optimal series of such events in a canonical order can be obtained in *O
 MathType@MTEF@5@5@+=feaafiart1ev1aaatCvAUfKttLearuWrP9MDH5MBPbIqV92AaeXatLxBI9gBamrtHrhAL1wy0L2yHvtyaeHbnfgDOvwBHrxAJfwnaebbnrfifHhDYfgasaacH8akY=wiFfYdH8Gipec8Eeeu0xXdbba9frFj0=OqFfea0dXdd9vqai=hGuQ8kuc9pgc9s8qqaq=dirpe0xb9q8qiLsFr0=vr0=vr0dc8meaabaqaciaacaGaaeqabaWaaeGaeaaakeaaimaacqWFoe=taaa@383D@(*n*^2^) *time*.

*Proof*. As discussed above, Algorithm Sorting-by-ffbi transforms *α *into *I *using the minimum number of fusions, fissions and block-interchanges. Next, we follow to analyze its time-complexity. Notice that given an undirected graph with *p *vertices and *q *edges, all the connected components in this graph can be found in O
 MathType@MTEF@5@5@+=feaafiart1ev1aaatCvAUfKttLearuWrP9MDH5MBPbIqV92AaeXatLxBI9gBamrtHrhAL1wy0L2yHvtyaeHbnfgDOvwBHrxAJfwnaebbnrfifHhDYfgasaacH8akY=wiFfYdH8Gipec8Eeeu0xXdbba9frFj0=OqFfea0dXdd9vqai=hGuQ8kuc9pgc9s8qqaq=dirpe0xb9q8qiLsFr0=vr0=vr0dc8meaabaqaciaacaGaaeqabaWaaeGaeaaakeaaimaacqWFoe=taaa@383D@(*p *+ *q*) time using depth-first search or breadth-first search [31]. As a result, Step 1 can be done in O
 MathType@MTEF@5@5@+=feaafiart1ev1aaatCvAUfKttLearuWrP9MDH5MBPbIqV92AaeXatLxBI9gBamrtHrhAL1wy0L2yHvtyaeHbnfgDOvwBHrxAJfwnaebbnrfifHhDYfgasaacH8akY=wiFfYdH8Gipec8Eeeu0xXdbba9frFj0=OqFfea0dXdd9vqai=hGuQ8kuc9pgc9s8qqaq=dirpe0xb9q8qiLsFr0=vr0=vr0dc8meaabaqaciaacaGaaeqabaWaaeGaeaaakeaaimaacqWFoe=taaa@383D@(*n*^2^) time for computing the connected components in the induced bipartite graph G
 MathType@MTEF@5@5@+=feaafiart1ev1aaatCvAUfKttLearuWrP9MDH5MBPbIqV92AaeXatLxBI9gBamrtHrhAL1wy0L2yHvtyaeHbnfgDOvwBHrxAJfwnaebbnrfifHhDYfgasaacH8akY=wiFfYdH8Gipec8Eeeu0xXdbba9frFj0=OqFfea0dXdd9vqai=hGuQ8kuc9pgc9s8qqaq=dirpe0xb9q8qiLsFr0=vr0=vr0dc8meaabaqaciaacaGaaeqabaWaaeGaeaaakeaaimaacqWFge=raaa@382D@(*α*, *I*), since in the worst case, the number of edges in G
 MathType@MTEF@5@5@+=feaafiart1ev1aaatCvAUfKttLearuWrP9MDH5MBPbIqV92AaeXatLxBI9gBamrtHrhAL1wy0L2yHvtyaeHbnfgDOvwBHrxAJfwnaebbnrfifHhDYfgasaacH8akY=wiFfYdH8Gipec8Eeeu0xXdbba9frFj0=OqFfea0dXdd9vqai=hGuQ8kuc9pgc9s8qqaq=dirpe0xb9q8qiLsFr0=vr0=vr0dc8meaabaqaciaacaGaaeqabaWaaeGaeaaakeaaimaacqWFge=raaa@382D@(*α*,*I*) is *χ*(*α*) × *χ*(*I*) and *χ*(*α*) = O
 MathType@MTEF@5@5@+=feaafiart1ev1aaatCvAUfKttLearuWrP9MDH5MBPbIqV92AaeXatLxBI9gBamrtHrhAL1wy0L2yHvtyaeHbnfgDOvwBHrxAJfwnaebbnrfifHhDYfgasaacH8akY=wiFfYdH8Gipec8Eeeu0xXdbba9frFj0=OqFfea0dXdd9vqai=hGuQ8kuc9pgc9s8qqaq=dirpe0xb9q8qiLsFr0=vr0=vr0dc8meaabaqaciaacaGaaeqabaWaaeGaeaaakeaaimaacqWFoe=taaa@383D@(*n*) and *χ*(*I*) = O
 MathType@MTEF@5@5@+=feaafiart1ev1aaatCvAUfKttLearuWrP9MDH5MBPbIqV92AaeXatLxBI9gBamrtHrhAL1wy0L2yHvtyaeHbnfgDOvwBHrxAJfwnaebbnrfifHhDYfgasaacH8akY=wiFfYdH8Gipec8Eeeu0xXdbba9frFj0=OqFfea0dXdd9vqai=hGuQ8kuc9pgc9s8qqaq=dirpe0xb9q8qiLsFr0=vr0=vr0dc8meaabaqaciaacaGaaeqabaWaaeGaeaaakeaaimaacqWFoe=taaa@383D@(*n*). In Step 2, there are *ω *outer iterations, each computing the minimum series of events needed to transform *β*_*i *_into *J*_*i*_, where 1 ≤ *i *≤ *ω*. Clearly, Steps 2.1 costs O
 MathType@MTEF@5@5@+=feaafiart1ev1aaatCvAUfKttLearuWrP9MDH5MBPbIqV92AaeXatLxBI9gBamrtHrhAL1wy0L2yHvtyaeHbnfgDOvwBHrxAJfwnaebbnrfifHhDYfgasaacH8akY=wiFfYdH8Gipec8Eeeu0xXdbba9frFj0=OqFfea0dXdd9vqai=hGuQ8kuc9pgc9s8qqaq=dirpe0xb9q8qiLsFr0=vr0=vr0dc8meaabaqaciaacaGaaeqabaWaaeGaeaaakeaaimaacqWFoe=taaa@383D@(*n*_*i*_) time for computing *J*_*i*_βi−1
 MathType@MTEF@5@5@+=feaafiart1ev1aaatCvAUfKttLearuWrP9MDH5MBPbIqV92AaeXatLxBI9gBaebbnrfifHhDYfgasaacH8akY=wiFfYdH8Gipec8Eeeu0xXdbba9frFj0=OqFfea0dXdd9vqai=hGuQ8kuc9pgc9s8qqaq=dirpe0xb9q8qiLsFr0=vr0=vr0dc8meaabaqaciaacaGaaeqabaqabeGadaaakeaaiiGacqWFYoGydaqhaaWcbaGaemyAaKgabaGaeyOeI0IaeGymaedaaaaa@31B9@ and Step 2.2 takes only a constant time. The time cost of Step 2.3 is mostly contributed from that of Step 2.3.3. There are *n*_*fu *_inner iterations in Step 2.3, each with the purpose of finding two numbers *x *and *y *that are both in the same cycle in *γ *and, however, in the different cycles in *β*_*i*_. In the worst case, Step 2.3 needs *n*_*i*_ find-set operations and *n*_*fu *_union operations to finish its overall process. Note that Step 2.3.1 can be implemented by initially creating a set for each number in gene(*β*_*i*_) and then performing *n*_*i *_- *χ*(*β*_*i*_) union operations to generate *χ*(*β*_*i*_) sets with each corresponding to a cycle in *β*_*i*_, where *χ*(*β*_*i*_) = *n*_*fu *_+ 1. Hence, the total number of union operations is *n*_*i *_- 1 in Step 2.3. In fact, these find-set and union operations can be implemented in O
 MathType@MTEF@5@5@+=feaafiart1ev1aaatCvAUfKttLearuWrP9MDH5MBPbIqV92AaeXatLxBI9gBamrtHrhAL1wy0L2yHvtyaeHbnfgDOvwBHrxAJfwnaebbnrfifHhDYfgasaacH8akY=wiFfYdH8Gipec8Eeeu0xXdbba9frFj0=OqFfea0dXdd9vqai=hGuQ8kuc9pgc9s8qqaq=dirpe0xb9q8qiLsFr0=vr0=vr0dc8meaabaqaciaacaGaaeqabaWaaeGaeaaakeaaimaacqWFoe=taaa@383D@(*n*_*i*_) time using the so-called "static disjoint set union and find" algorithm proposed by Gabow and Tarjan [32]. In other words, Step 2.3 can cost only O
 MathType@MTEF@5@5@+=feaafiart1ev1aaatCvAUfKttLearuWrP9MDH5MBPbIqV92AaeXatLxBI9gBamrtHrhAL1wy0L2yHvtyaeHbnfgDOvwBHrxAJfwnaebbnrfifHhDYfgasaacH8akY=wiFfYdH8Gipec8Eeeu0xXdbba9frFj0=OqFfea0dXdd9vqai=hGuQ8kuc9pgc9s8qqaq=dirpe0xb9q8qiLsFr0=vr0=vr0dc8meaabaqaciaacaGaaeqabaWaaeGaeaaakeaaimaacqWFoe=taaa@383D@(*n*_*i*_) time. By the same principle, it can be verified that the time cost of Step 2.4 is O
 MathType@MTEF@5@5@+=feaafiart1ev1aaatCvAUfKttLearuWrP9MDH5MBPbIqV92AaeXatLxBI9gBamrtHrhAL1wy0L2yHvtyaeHbnfgDOvwBHrxAJfwnaebbnrfifHhDYfgasaacH8akY=wiFfYdH8Gipec8Eeeu0xXdbba9frFj0=OqFfea0dXdd9vqai=hGuQ8kuc9pgc9s8qqaq=dirpe0xb9q8qiLsFr0=vr0=vr0dc8meaabaqaciaacaGaaeqabaWaaeGaeaaakeaaimaacqWFoe=taaa@383D@(*n*_*i*_). As for Step 2.5, adopted from our previous work [14], it takes O
 MathType@MTEF@5@5@+=feaafiart1ev1aaatCvAUfKttLearuWrP9MDH5MBPbIqV92AaeXatLxBI9gBamrtHrhAL1wy0L2yHvtyaeHbnfgDOvwBHrxAJfwnaebbnrfifHhDYfgasaacH8akY=wiFfYdH8Gipec8Eeeu0xXdbba9frFj0=OqFfea0dXdd9vqai=hGuQ8kuc9pgc9s8qqaq=dirpe0xb9q8qiLsFr0=vr0=vr0dc8meaabaqaciaacaGaaeqabaWaaeGaeaaakeaaimaacqWFoe=taaa@383D@(*n*_*bi*_*n*_*i*_) time, where  = O
 MathType@MTEF@5@5@+=feaafiart1ev1aaatCvAUfKttLearuWrP9MDH5MBPbIqV92AaeXatLxBI9gBamrtHrhAL1wy0L2yHvtyaeHbnfgDOvwBHrxAJfwnaebbnrfifHhDYfgasaacH8akY=wiFfYdH8Gipec8Eeeu0xXdbba9frFj0=OqFfea0dXdd9vqai=hGuQ8kuc9pgc9s8qqaq=dirpe0xb9q8qiLsFr0=vr0=vr0dc8meaabaqaciaacaGaaeqabaWaaeGaeaaakeaaimaacqWFoe=taaa@383D@*n*_*i*_). As a result, the time cost of Step 2 is O
 MathType@MTEF@5@5@+=feaafiart1ev1aaatCvAUfKttLearuWrP9MDH5MBPbIqV92AaeXatLxBI9gBamrtHrhAL1wy0L2yHvtyaeHbnfgDOvwBHrxAJfwnaebbnrfifHhDYfgasaacH8akY=wiFfYdH8Gipec8Eeeu0xXdbba9frFj0=OqFfea0dXdd9vqai=hGuQ8kuc9pgc9s8qqaq=dirpe0xb9q8qiLsFr0=vr0=vr0dc8meaabaqaciaacaGaaeqabaWaaeGaeaaakeaaimaacqWFoe=taaa@383D@(*ζn*), where *ζ *is the maximum *n*_*bi *_among all iterations in Step 2 and *ζ *<*n*. Clearly, Steps 3 and 4 cost constant time. Therefore, the total time-complexity of Algorithm Sorting-by-ffbi is O
 MathType@MTEF@5@5@+=feaafiart1ev1aaatCvAUfKttLearuWrP9MDH5MBPbIqV92AaeXatLxBI9gBamrtHrhAL1wy0L2yHvtyaeHbnfgDOvwBHrxAJfwnaebbnrfifHhDYfgasaacH8akY=wiFfYdH8Gipec8Eeeu0xXdbba9frFj0=OqFfea0dXdd9vqai=hGuQ8kuc9pgc9s8qqaq=dirpe0xb9q8qiLsFr0=vr0=vr0dc8meaabaqaciaacaGaaeqabaWaaeGaeaaakeaaimaacqWFoe=taaa@383D@(*n*^2^).

### Construction of orthologous genes

To analyze the rearrangement of three *Vibrio *genomes, we identified and constructed a table of orthologous genes that are putatively not involved in horizontal gene transfer (HGT) events by adopting the so-called symmetrical best hits (SymBets for short). In principle, two genes match and give SymBets if they are more similar to each other than they are to any other genes from the compared genomes [33,34]. Detection of such SymBet genes is, yet arguably, the simplest and most suitable method for identification of probable orthologs for closely related genomes [33,34]. Particularly, this prediction of orthologs holds to be applicable even when sequence similarity between the compared proteins is relatively low [33,34]. For the purpose of excluding paralogous genes derived from lineage-specific gene duplications, we here considered only one-to-one orthologous genes, which actually have been demonstrated as a major pattern in prokaryotic genome evolution [34]. Therefore, we used the following steps to identify an HGT-free table of one-to-one orthologous genes from the three complete *Vibrio *genomes. First, the GenePlot [35] program offered by NCBI was utilized to find and construct a table of SymBet genes between each pair of *Vibrio *genomes. Next, after removing all one-to-many or many-to-many SymBets, the three resulting tables of SymBet genes were joined to give a new one of one-to-one orthologous genes for all the three *Vibrio *genomes by using a rule as follows. If genes *a *(from genome *A*) and *b *(from genome *B*),*b *and *c *(from genome C), and *c *and *a *are all one-to-one SymBet pairs, then a, *b *and *c *are considered as one-to-one orthologous genes for the genomes *A*,*B *and *C*. In other words, the SymBet relationships among a, *b *and *c *result in a triangle. Finally, those genes that were involved in the putative HGT events detected and available in the Horizontal Gene Transfer Database [36] were then deleted from the table of one-to-one orthologous genes.

## Authors' contributions

CLL and HTC contributed equally to this work. CLL conceived of the study, participated in the design and analysis of algorithm and drafted the manuscript. YLH participated in the algorithm design and software development, and carried out the bioinformatics experiments. TCW participated in the software development. HTC participated in the design and coordination of this study as well as in drafting the manuscript.

## Note

^1^The Institute for Genome Research (TIGR) offers a website of Comprehensive Microbial Resource (CMR) that provides information on all of the publicly available and complete bacterial genomes.

## Figures and Tables

**Table 1:** The sequence information of three pathogenic *Vibrio *species, each with two circular chromosomes.

Accession NO.	Species	Chromosome	Size (Mbps)
[GenBank:NC_005139]	*V. vulnificus *YJ016	1	3.4
[GenBank:NC_005140]	*V. vulnificus *YJ016	2	1.9
[GenBank:NC_004603]	*V. parahaemolyticus *RIMD 2210633	1	3.3
[GenBank:NC_004605]	*V. parahaemolyticus *RIMD 2210633	2	1.9
[GenBank:NC_002505]	*V. cholerae *El Tor N16961	1	3.0
[GenBank:NC_002506]	*V. cholerae *El Tor N16961	2	1.0

**Table 2:** The calculated rearrangement distances among *V. vulnificus*, *V. parahaemolyticus *and *V. cholerae *by fusions, fissions and block-interchanges.

Species Compared	*V. vulnificus*	*V. parahaemolyticus*	*V. cholerae*
*V. vulnificus*	0	174	364
*V. parahaemolyticus*	174	0	391
*V. cholerae*	364	391	0

**Table 3:** The calculated distances among *V. vulnificus *[GenBank:X76333], *V. parahaemolyticus *[GenBank:X56580] and *V. cholerae *[GenBank:X76337] by the traditional comparative analysis of their 16S rRNA gene sequences (accession numbers of 16S rRNAs are given in square brackets).

Species Compared	*V. vulnificus*	*V. parahaemolyticus*	*V. cholerae*
*V. vulnificus*	0.000000	0.034524	0.050261
*V. parahaemolyticus*	0.034524	0.000000	0.076739
*V. cholerae*	0.050261	0.076739	0.000000
